# Mucosal immune response in biology, disease prevention and treatment

**DOI:** 10.1038/s41392-024-02043-4

**Published:** 2025-01-08

**Authors:** Xiaoxue Zhou, Yuchen Wu, Zhipeng Zhu, Chu Lu, Chunwu Zhang, Linghui Zeng, Feng Xie, Long Zhang, Fangfang Zhou

**Affiliations:** 1 School of Medicine, Hangzhou City University, Hangzhou, China; 2 MOE Laboratory of Biosystems Homeostasis & Protection and Innovation Center for Cell Signaling Network, Life Sciences Institute, Zhejiang University, Hangzhou, China; 3 The First School of Medicine, Wenzhou Medical University, Wenzhou, Zhejiang China; 4 The First Affiliated Hospital, the Institutes of Biology and Medical Sciences, Suzhou Medical College, Soochow University, Suzhou, Jiangsu China; 5 The MOE Basic Research and Innovation Center for the Targeted Therapeutics of Solid Tumors, The First Affiliated Hospital, Jiangxi Medical College, Nanchang University, Nanchang, China; 6 Cancer Center, Zhejiang University, Hangzhou, Zhejiang China

**Keywords:** Immunological disorders, Vaccines

## Abstract

The mucosal immune system, as the most extensive peripheral immune network, serves as the frontline defense against a myriad of microbial and dietary antigens. It is crucial in preventing pathogen invasion and establishing immune tolerance. A comprehensive understanding of mucosal immunity is essential for developing treatments that can effectively target diseases at their entry points, thereby minimizing the overall impact on the body. Despite its importance, our knowledge of mucosal immunity remains incomplete, necessitating further research. The outbreak of severe acute respiratory syndrome coronavirus 2 (SARS-CoV-2) has underscored the critical role of mucosal immunity in disease prevention and treatment. This systematic review focuses on the dynamic interactions between mucosa-associated lymphoid structures and related diseases. We delve into the basic structures and functions of these lymphoid tissues during disease processes and explore the intricate regulatory networks and mechanisms involved. Additionally, we summarize novel therapies and clinical research advances in the prevention of mucosal immunity-related diseases. The review also addresses the challenges in developing mucosal vaccines, which aim to induce specific immune responses while maintaining tolerance to non-pathogenic microbes. Innovative therapies, such as nanoparticle vaccines and inhalable antibodies, show promise in enhancing mucosal immunity and offer potential for improved disease prevention and treatment.

## Introduction

The human body operates as a perpetual battleground where countless microorganisms perpetually vie for viability. At the forefront of this ceaseless conflict reside the mucosal surfaces, encompassing almost all the linings of the interface between the internal and external environment. These mucosal territories function both as protective barriers and as gateways for various pathogens, thus necessitating a robust initial line of defense. Armed with a dynamic array of mechanisms, mucosal immunity stands as the sentinel of these portals, engaging not only in the fight against infectious agents but also in the nuanced management of the diverse commensal microorganisms inhabiting these surfaces. Mucosal immunity is a cornerstone in the realm of immunology, and it warrants a profound exploration. While the current global crisis, COVID-19 has thrust mucosal immunity into the spotlight, it is imperative to appreciate its significance in a broader context.

The imperative to delve into mucosal immunity arises from several critical factors. Firstly, mucosal surfaces are the primary entry points for a myriad of pathogens, making them pivotal in the initial immune response. Understanding these mechanisms is crucial for developing effective preventative and therapeutic strategies against infectious diseases. Secondly, the mucosal immune system operates with a delicate balance, distinguishing between harmful pathogens and benign commensals, which is essential for maintaining homeostasis and preventing chronic inflammatory conditions. Thirdly, with the ongoing COVID-19 pandemic, there has been an unprecedented focus on respiratory mucosal immunity, highlighting the need for a deeper understanding to combat current and future respiratory pathogens. Lastly, advancements in mucosal immunology have the potential to revolutionize vaccine delivery and immune therapies, providing targeted and efficient solutions to a range of diseases. Thus, exploring the complexities of mucosal immunity is not only scientifically enriching but also of paramount importance for public health.

Here we review to elucidate the multifaceted dimensions of mucosal immunity, highlighting its pivotal roles in countering a wide spectrum of mucosal-related diseases and conditions. This review commences with an in-depth examination of mucosal front-line immunity to appreciate its broader significance. We furnish a brief overview of mucosal immunity and SARS-CoV-2, emphasizing the connection between the two sides and the necessity to familiarize them. Next, we venture into the localized wars waged within the mucosal terrain including the respiratory tract and the gastrointestinal tract, focusing on the structure of mucosal-associated lymphoid tissues and their role against invading pathogens. Subsequently, we present the intricate mucosal immune signaling networks orchestrating responses to mucosal threats, exploring both initiation and regulation. Moreover, we delve into the examination of prevention and treatment strategies rooted in mucosal immunity, offering insights into innovative approaches, such as mucosal vaccines, inhalable antibodies, and novel preventions and treatments with broader implications beyond the pandemic. Meanwhile, in the post-pandemic era, the aftermath of the disease has raised questions about lingering symptoms and long-term health effects, adding another layer of complexity to our understanding of mucosal immunity’s role in both acute and chronic conditions. In all, this review endeavors to cast a discerning eye upon the world of mucosal immunity, reminding its multifaceted significance beyond the immediate challenges posed by COVID-19.

## An overview of the frontline mucosal immune system

Mucus, which mainly contains lipids, secretory proteins, and commensal microbiota, is distributed thAccording to previouroughout the body and is directly exposed to pathogens and toxic agents. In adults, the skin surface area is approximately 2 m^2^; however, mucosal surface area may exceed 400 m^2.^^1^ The mucosal surface is broad enough to be a site of enormous immune reactions and habitation for commensal microorganisms. Therefore, the mucosal immune system constitutes the largest portion of the immune system, including both innate and adaptive immunity.^2^ Physically, the mucosal immune system protects the host from foreign pathogenic microorganisms and viruses, and harmful substances. If foreign pathogens and harmful substances penetrate the mucosal surface, the mucosal immune system instantly initiates an immune response to recognize and neutralize them. In addition to immune surveillance and defense, the mucosal immune system plays a significant role in maintaining immune tolerance.^3^ The gastrointestinal tract is in contact with food every day and is the largest habitat for human microbes. Various foods and the commensal microbiota are exogenous, which in principle, should be rejected by the immune system. The mucosal immune system in the gastrointestinal tract establishes and maintains immune tolerance to innocuous foreign antigens to ensure the exchange and absorption of beneficial substances.^3,4^

Due to the presence of clinical symptoms, including fever, cough, sputum production, dyspnea, and headache in COVID-19 patients and accumulated experience with other coronaviruses, such as the Middle East respiratory syndrome coronavirus and SARS-CoV-1,^5,6^ SARS-CoV-2 was naturally regarded as a respiratory virus from the onset. However, growing evidence has revealed that SARS-CoV-2 also attacks other areas in the body, such as the intestinal tract, heart, kidney, liver, mammary gland, eyeball, and brain in humans,^7–14^ suggesting that it is more than a respiratory virus. Moreover, the virus causes extended damages to more tissues and organs due to induced systemic immune-mediated responses and inflammation, as opposed to direct infection. SARS-CoV-2 invades cells principally via angiotensin-converting enzyme-2 (ACE2) and transmembrane protease serine 2 (TMPRSS2) on cell surfaces.^15^ Spike (S) protein is primed by TMPRSS2 and binds to the ACE2 entry receptor during infection. Given that ACE2 is widely expressed in most epithelial cells, SARS-CoV-2 can successfully infect various organs.^16,17^ According to previous studies, the S protein is necessary for SARS-CoV-2 invasion of the host cells,^18^ and the toxicity and infectivity of different variants and subvariants chiefly depend on the mutations in the gene encoding the S protein.^19,20^ Amino acid replacement and changes in the S protein structure affect SARS-CoV-2 invasive ability, incurring the loss of efficacy of the original antibodies;^21,22^ thus, superinfection is a concern in COVID-19. Extensive investigations of S protein-mediated interactions between the virus and host cells are ongoing, and numerous treatments, drug designs, and vaccine developments for COVID-19 are targeted at the S protein. The mucosal immune system initiates several defensive measures during all stages of SARS-CoV-2 attacks against sensitive organs and even aids the recovery of the body post-COVID-19.^23^ However, virus strives to evade or even destroy the immune surveillance and immune responses initiated no matter by the mucosal immune system or the systemic immune system.

In this review, to clarify these complicated interplays in context, we summarized representative cells and components of the mucosal immune system and described their associations with SARS-CoV-2 clinically and experimentally. Further, we explored efficacious and innovative anti-virus measures with respect to the mucosal immune system to provide insights into COVID-19 treatment.

**Fig. 1:**
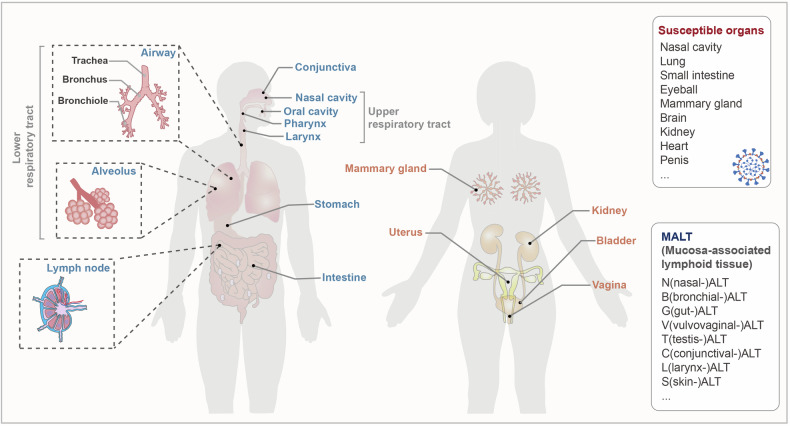
Distribution of the mucosal immune system in the body. The mucosal immune system is distributed throughout the body primarily in the respiratory, gastrointestinal, and urogenital tracts. The respiratory tract includes, in ascending order, the nasal cavity, oral cavity, airway, and lungs. The airway is divided into the trachea, bronchi, and bronchioles according to branching of the airway. The lungs comprise mainly the alveolus and pulmonary interstitium. The gastrointestinal tract, particularly the intestine, is dotted with lymph nodes, and the mucosal immune response is the most active immune response in the gastrointestinal tract. The uterus, bladder, and vagina comprise the female urogenital tract. In addition, the mucosal immune system is distributed in other important regions, such as the conjunctiva, middle ear, and breast. The most important mucosal immune component, mucosa-associated lymphoid tissue can be divided according to the organs they occupy, and the susceptible organs for SARS-CoV-2 widely exist throughout the body

In terms of distribution, the mucosal immune system is mainly present in the upper respiratory, lower respiratory, gastrointestinal, and urogenital tracts (Fig. 1). Moreover, the mucosal immune system is present in other areas with mucosal tissues, such as the conjunctival and lacrimal glands of the eye, middle ear, salivary glands, and mammary glands. Respiratory virus infection routes correlated highly with mucosal immune system distribution,^7,12,13,24,25^ which indicates their close relationship.

Fully covered by mucus and mucus-secreting epithelium, the interface of the external and internal environments is well protected by the mucosal immune system.^26^ Mucosa-associated lymphoid tissue (MALT) is present in the spaces between epithelial cells and mainly beneath the epithelium layer.^27^ Lymph and blood capillaries are distributed around the epithelial layer and MALT, through which bidirectional immune cell exchanges occur.^28^ This suggests that certain immune cells circulate in the body instead of being tissue-resident, ensuring communication between the mucosal immune system and the central immune system. Thus, mucus, the mucus-secreting epithelium, and MALT are the core components of the mucosal immune system. Each performs its own functions and interrelates with the whole immune system when confronting respiratory virus infection. In this review, we also examined and organized the influence of respiratory virus and its major immune-mediated reactions in three different structural compositions.

In the MALT, which lies in submucosal membrane sites, immune cells are accumulated and activated to initiate mucosal immunity. The primary functions of MALT are to produce immunoglobulin A (IgA) and induce T-helper 2 cells (Th2)-dependent reactions.^27^ MALT mainly contains nasopharyngeal-associated lymphoid tissue in the upper respiratory tract, bronchial-associated lymphoid tissue in the lower respiratory tract, gut-associated lymphoid tissue in the gastrointestinal tract, and vulvovaginal-associated lymphoid tissue /testis-associated lymphoid tissue in the urogenital tract. Anatomically, MALT can be divided into organized and diffuse mucosal lymphoid tissues.

Compared with organized mucosal lymphoid tissues, the distribution of immune cells in diffuse mucosal lymphoid tissues is not well-defined or regular. Intraepithelial lymphocytes (IELs) and the lamina propria (LP) are major diffuse mucosal lymphoid tissues. IELs may be the first lymphocytes to encounter respiratory virus that invades the epithelial tissue. Increased mucosal infiltration with IELs has been observed in the small intestine of patients with COVID-19,^29^ which offers a potential explanation for the gastrointestinal symptoms associated with SARS-CoV-2 infection. The LP is a thin layer of loose connective tissue beneath the epithelium, which is rich in mature plasma cells and macrophages and serves as an auxiliary site for antibody secretion. Secreted antibodies play a vital role in the adaptive immunity initiated by the mucosal immune system. DCs and other APCs present major histocompatibility complex II-antigens to activate CD 4^+^ T cells, while essential cytokines assist naïve B cells in differentiating into plasma cells, and then secrete large amounts of antibodies.

## Local wars between the mucosal immune system and pathogens

Methods for respiratory virus to infect, transmit and escape are multifarious, and immune responses from the mucosal immune system are also all-round to cope with them. To place these relations in context, we focus on three significant epicenters: the upper respiratory tract (URT), the lower respiratory tract (LRT) and the gastrointestinal tract, where interactions between the mucosal immune system and SARS-CoV-2 are complex. Here, we detailly portray structures and functions of mucosal tissue in these regions, collate various mechanisms and characteristics of immune responses, and expound regional similarity and heterogeneity for COVID-19.

### Characteristics of upper respiratory tract mucosal immunity

#### Structure and mucosal immune microenvironment of the upper respiratory tract

**Fig. 2:**
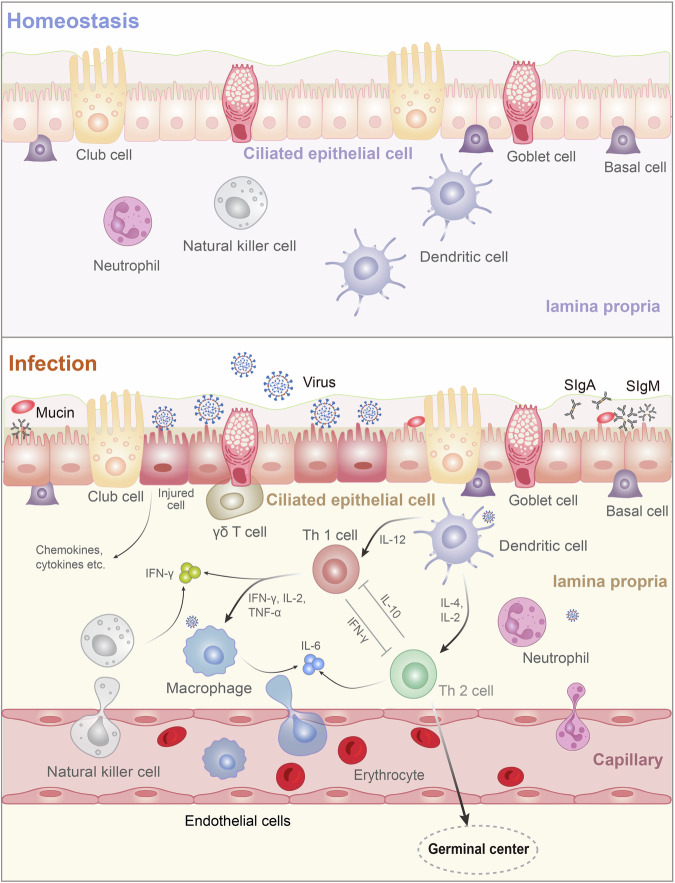
Mucosal immune system in the upper respiratory tract. The mucosal immune system in the airway mainly includes a ciliated epithelial cell layer in which club and goblet cells are the main mucosal secretory cells that secrete mucin and mucosal lipids. Basal cells are epithelial stem cells that renew and supplement epithelial cells. During SARS-CoV-2 invasion, T cells are activated and natural killer cells, macrophages, and neutrophils pass through the endothelial cells of the capillaries to the lamina propria to perform their immune functions. DCs secrete IL-12 to activate Th1 cells, IL-2 and IL-4 to activate Th2 cells respectively. Th1 and Th2 exhibit an antagonistic relationship. IFN-γ secreted by NK cells and Th1 cells can promote cellular antiviral response. Activated Th2 cells play a more important role in the germinal center. IL-6 mainly produced by macrophages and Th2 cells is a significant biomarker for severe COVID-19. The cilia can cross the mucosal layer to contact SARS-CoV-2, and the cilia surface can express angiotensin converting enzyme-2 and transmembrane protease serine 2; thus, they can transport SARS-CoV-2 directly. Ciliary dyneins can transfer SARS-CoV-2 to the cell surface and infect the cells through receptors on the cell surface. After infection, the PAK 1/4 pathway activates the reprogramming of cytoskeletal proteins in the microvilli, making the microvilli longer and larger. Reprogrammed microvilli can extend more viral particles to the mucus layer, which is prone to viral transmission

URT is the first to bear the brunt once pathogens are inhaled, and early control of respiratory virus infection and suppression of its transmission primarily lean on robust mucosal immune responses in URT^30,31^(Fig. 2). NALT represents an important induction site to generate mucosal immunity. Without accurate locations in non-rodent mammals, NALT can be lymphoid follicles or other aggregates of lymphocytes in the nasal cavity,^32^ and it is considered analogous to Waldeyer’s ring in humans.^33^ Waldeyer’s ring (also known as the tonsils) is lymphatic masses located in nasopharyngeal and oral cavities, which mainly comprises the palatine tonsils, nasopharyngeal tonsil (adenoid) and lingual tonsil, with the tubal tonsils and lateral pharyngeal bands playing a minor role. Serving as the front immune outposts, the tonsils surveil and filter most pathogens once the inhaled or ingested external substances enter nasopharyngeal or oral cavities. Typically, the nasopharyngeal tonsil sits on the roof and posterior wall of the nasopharynx, and like other tonsil glands, crypt epithelium and lymphoid follicles are their specialized immune compartments.

Crypts are generated because stratified squamous epithelium subsides into the underlying lymphoid tissue.^34^ These invagination structures considerably expand the tonsillar surface area and make it easier to contact and hide pathogenic bacteria and allergens. To deal with abundant pathogens, crypts own powerful immune defense independent of the germinal center (GC). Apart from squamous epithelial cells, immune cells including T cells, dendritic cells (DCs), natural killer (NK) cells, and microfold (M) cells infiltrate the mesenchymal layer beneath the epithelium or exist on the surface.^35^ Lymphocytes from the nasopharyngeal tonsil can also migrate to the mucus, and this process is active and selective.^36^ Here we talk about T and NK cells, and the remaining will be discussed later. T cells infiltrating the epithelium can be divided into two subtypes owing to different T-cell receptors (TCR), αβ T cells and γδ T cells. αβ T cells are classic and well-known, most of which (80%) are CD4+ subsets.^37^ Among them T-helper 1 cells (Th1) possess the property of cytokine production like interferon (IFN)-γ, tumor necrosis factor (TNF)-α and interleukin (IL)-2 to activate macrophages against intracellular pathogens,^38,39^ and overactivation of Th1 will induce inflammatory-related autoimmune diseases such as type-1 diabetes (T1D), rheumatoid arthritis (RA), and delayed-type hypersensitivity responses.^40^ T-helper 2 cells (Th2) release IL-4 and IL-5 to activate eosinophils, mast cells and IgE secretion of B cells against extracellular pathogens.^41^ Adenoid hypertrophy (AH), usually caused by passive smoking and allergic rhinitis, is one of the most common symptoms of adenoids in children.^42,43^ AH will bring lots of complications like otitis media, obstructive sleep apnea/hypopnea syndrome and chronic nasal obstruction, because the enlarged adenoid constricts other tissues or tracts. AH may lead to immune disorder of adenoid due to abnormal differentiation of T cells, and the dynamic alternation between Th1 and Th2 cells suggests different situations of AH. Th2 cells decline in children undergoing adenoidectomies for otitis media,^39^ while in recurrent cases, cytokines from Th1 are lower. However, there is also a significant reduction of Th1 cells in COVID-19 patients who have high inflammatory indexes.^44^ IFN-γ secreted by Th1 cells and IL-10 secreted by Th2 can inhibit the polarization of the other,^45,46^ so Th1 and Th2 cells antagonize each other, and the homeostasis between Th1 and Th2 is vital to the whole immune system.^47^ γδ T cells only take up a small portion (2–3%) but are also important.^37^ They belong to intraepithelial lymphocytes and mostly scatter in the epithelial layer and exhibit various cytotoxic activities and repair capabilities to keep immune surveillance and steady-state tissue physiology.^48,49^ In patients with hypertrophic obstructive adenoids, the percentage of γδ T cells decreasing and the recruitment of neutrophils reducing suggest that γδ T cells assist in maintaining the integrity of the adenoids epithelium.^50,51^ γδ T cells were reported to inhibit the replication of SARS-CoV-2,^52^ but their number is prone to lower in patients with COVID-19;^52–55^ moreover, at most 8.5% of the components in the adenoid mucus secretion are γδ T cells,^56^ thus the role of γδ T cells in mucosal immunity is worth thinking about. Unlike NK cells from peripheral blood, adenoid-derived NK cells are CD16- and lack perforin.^57,58^ They are more like Th1 cells, prone to produce IFN-γ and also decreasing in pediatric adenoid hypertrophy cases.^59,60^

Lymphoid follicles have structures similar to those of lymph nodes. Dominant B cells proliferate and differentiate in germinal centers, whereas minor T cells and DCs occupy the interfollicular areas.^37^ Additionally, other organized mucosal lymphoid tissues, such as isolated lymphoid follicles are widely distributed in the gastrointestinal tract and possess the ability to produce antibodies. B cells in lymphoid follicles of adenoids can more swiftly react to product antibodies than others.^61^ On the one hand, the location of adenoids is unique; the earliest antigen recognition and rapid immune signaling help activate B cells in GC. For another, follicular T helper (T_FH_) cells boom naive B cells to produce immunoglobulins via IL-21 secretion,^62,63^ and in turn B cells express B cell lymphoma 6 regulating naïve T cell differentiation into T_FH_ cells;^64–66^ this loop strengthens the antibody production capacity of adenoids. Single-cell sequencing data indicates that T_FH_ cell populations are expanded after COVID-19, suggesting their role in the generation and persistence of SARS-CoV-2-specific GC responses.^67^

#### Respiratory mucosa Cilia-mediated accelerated infection

Mucus-secreting epithelium and the LP collectively refer to the mucous membranes. Mucus-secreting epithelium is involved in the production of essential mucosal components and the mediation of substance transportation. Epithelial cells form a scaffold of mucus-secreting epithelial tissue. Epithelial cells have several functional subtypes that are important sensors and reactors to infection and inflammation.^68^ In addition, epithelial and goblet cells secrete surfactant, complement proteins, mucins, and antimicrobial peptides (AMPs), which are the primary mucosal innate immune elements.^26,68^ Basal cells, known as epithelial stem cells, divide and differentiate into epithelial cells for cell renewal and supplementation.

Despite numerous specific and non-specific anti-virus measures taken by the mucosal immune system, epithelial cells are likely to be infected, which may be induced by the motile cilia of the epithelial cells. Regarding the microenvironment of ciliated epithelial cells, the underlying periciliary layer separates mucus and the epithelia, and the apertures of the periciliary layer are too small (~25 nm) to permit large virus (~100 nm) to penetrate and thus gain access to the epithelia.^69^ However, SARS-CoV-2 can overcome this physical barrier and infect ciliated epithelial cells. Moreover, nasal ciliated epithelial cells are the primary spots where virus replicates during the early stages of COVID-19.^70^ Wu, C.T., et al. found that motile cilia give rise to large-scale infections in ciliated epithelial cells.^71^ Motile cilia are long and slim enough to penetrate the periciliary layer,^69^ and ACE2 and TMPRSS2, two vital factors for virus entry, are expressed on motile cilia surface.^72,73^ Therefore, virus can directly contact and infect motile cilia, or driven by ciliary dynein, adhered virus particles move to the cell body from the tip of the cilia, and on the cell surface, virus enters the cell through receptors. Moreover, SARS-CoV-2 regulates p21-activated kinases 1 and 4 to reprogram the microvilli, thereby facilitating microvillus elongation and viral egression, which accelerates virus budding and transmission.^71^

Apart from conventional epithelial cell components, dendrites of the olfactory receptor neurons stretch to the mucus of the nasal epithelium to sense odor molecules and transmit odorant signals to the central nervous system. Meinhardt J. et al. first reported that coronavirus penetrated the interface of olfactory mucosa, doing harm to endothelial and nervous tissue at vicinities.^74^ Range of infection and destruction eventuates transient or even persistent smell loss in COVID-19 patients.^75^ More seriously, coronavirus reaches the brain;^76^ afterwards astrocytes could be infected,^77^ neuron and glia fusion causing loss of neuronal activity,^78,79^ and brain inflammation occurs.^80^

#### Non-specific antiviral functions of small mucosal components

Thus far, we have identified mucosal components, all of which demonstrate varying degrees of anti-coronavirus abilities. We generally discuss mucosal complement proteins, mucins, and AMPs here.

Enhancing both innate and adaptive immunity, the complement system is a conserved immune system throughout evolution.^81^ Complement proteins can attack the membranes of pathogenic bacteria, resulting in cytolysis, and gather antibodies and antigens to form adhesive immune complexes, thereby improving the endocytic efficiency of phagocytic cells. Mechanistically, the complement system can be activated through the classical, lectin, and alternative pathways. Booming complement activation is a distinctive characteristic compared to non-COVID respiratory failure, and the alternative pathway is most prevalent clinically.^82^ Through competing with factor H, a negative regulator of the complement system, S protein binds with heparan sulfate to dysregulate the alternative pathway.^83,84^ Moreover, S and nucleocapsid (N) proteins directly initiate the activation of the lectin pathway.^85^ IgG and IgM binding to the receptor-binding domain (RBD) of the S protein leads to the activation of the classical pathway,^86^ which is known as antibody-mediated complement-dependent cytotoxicity (CDC). The level of complement protein may be referred to as clinical severity and a risk factor for death. Critically ill patients tend high levels of C5a, C5b-9 as well as C3.^87,88^ Similarly, a survey from the UK Biobank shows that factor H and complement component 4-binding protein-α are correlated to morbidity.^89^ Therefore, complement-inhibiting ideas are advocated to apply in adjuvant remedies.^90,91^ However, studies have focused more on complement proteins in blood circulation than those in the mucus. Epithelial cells have the capability to produce complement proteins such as C1, C3, and C5,^92,93^ and according to transcriptome analysis, they are alternative sources for complement proteins except for the liver.^94^ This suggests that when coronavirus encounters the mucus, it is likely to be recognized by complement proteins. In some other respiratory diseases, the complement system in the mucosal surface has been reported. For example, the whole-genome expression analysis for mucosal samples of subjects with allergic rhinitis showed upregulation of the alternative pathway (factor P and C5aR).^95^ Interactions between complement proteins and coronavirus are more pronounced in the mucus because of higher viral concentrations, and research on mucosal complement proteins is necessary.

Mucins are a family of glycosylated proteins whose key functions are to lubricate the membrane surface and keep it moist. As part of mucosal immunity, mucins can bind to harmful microorganisms or recruit anti-microbial proteins to inhibit the colonization and reproduction of harmful microorganisms and consequently preserve a benign environment for commensal microbiota.^96^ Mucins also have potential anti- coronavirus effects. Mucin (MUC) 1 expression increases in severe COVID-19,^97^ suggesting that infection induces a stress response that diminishes infection. When MUC1, MUC4, and MUC21 are overexpressed, the cells become highly resistant to coronavirus.^98^ Moreover, Smet A. et al. created a set of multifaceted blood mucin mRNA signatures to assess the COVID-19 severity.^99^ Using the genome-wide bidirectional clustered regularly interspaced short palindromic repeat screens, Biering S.B., et al. identified a membrane-tethered mucin that restricted S protein-mediated entry.^100^ However, some mucins play apparently opposite roles; kidney injury molecule-1/T cell Ig mucin-1, a transmembrane protein expressing in epithelium of the lung and kidney, mediate coronavirus entry into cells as alternative receptors.

As another important component of the innate immunity in the mucosal immune system, AMPs have demonstrated antiviral activity against coronaviruses.^101^ They are small molecular peptides involved in innate immunity that generally attack and kill bacteria, yeasts, fungi, viruses, and even cancer cells directly. Specifically secreted by intestinal Paneth cells, human α defensin 5 is the predominant α defensin that competitively binds to ACE2 to inhibit coronavirus invasion.^102^ Owing to their simple sequences and extensive resistance against pathogens, AMPs have become important in novel drug research. Researchers generally screen natural AMPs and artificially combine them to integrate their advantages. Zhao, H.J. et al. designed an AMP, 4H30, based on human beta-defensin 2, which could play three anti- coronavirus roles: binding to the S protein to block entry, inhibiting endosomal acidification to block membrane fusion, and cross-linking virus particles with glycosaminoglycans to block replication.^103^ DP7, another designed AMP, has potent activity against the S protein entry.^104^ Not only can they directly resist coronavirus, but as adjuvants, AMPs can adjust adaptive immunity against the virus.^105^ In addition to focusing on the individual anti- coronavirus effects of each component, their collaborative effects are also worth exploring.

#### Structure and immune microenvironment of nasal-associated lymphoid tissue (NALT)

The human nasopharyngeal cavity contains nasal-associated lymphoid tissue (NALT), including the well-organized NALT (o-NALT) and diffuse NALT (d-NALT), the latter of which has been underexplored. Human o-NALT consists of a series of tonsils (palatine, nasopharyngeal and lingual tonsils). The tonsils are arranged in a circular pattern in the oropharyngeal cavity, forming the Waldeyer ring.^106^ In contrast, teleost fish possess the most ancient d-NALT discovered thus far and lack o-NALT,^107^ making them a good animal model for studying the response of d-NALT to pathogens.^108^ Teleost NALT comprises B cells scattered in the olfactory epithelium, with 48.5% being IgM+ B cells and 51.5% being IgT+ B cells.^107^ Additionally, there are two distinct CD8α + T cell populations in the lateral sensory epithelium and one in the apical mucosal epithelium,^109^ which collectively protect the olfactory organs of teleost fish from waterborne pathogens.^110^ However, a 2022 study has identified o-NALT in rainbow trout, located in the epithelium. This o-NALT exhibits a germinal center (GC) response akin to that in mammals. However, further studies are necessary to confirm the generality of that finding in teleost fish.^111^

Rodents possess o-NALT on both sides of the nasopharyngeal canal and d-NALT in the nasal cavity. Rodent NALT is considered as a functional analogue of human tonsils,^112^ often referred to as o-NALT, and is currently more extensively studied. Murine NALT develops postnatally, stimulated by environmental antigens.^33^ Murine NALT comprises high endothelial venules (HEVs), follicle-associated epithelium (FAE), T- and B-cell-enriched areas and antigen-presenting cells (APCs). M cells, located in FAE, are responsible for acquiring antigens from airway mucosal surfaces.^33^ M cells can endocytose antigens specifically or non-specifically and release them at the base of M cells, which facilitates the function of APCs. HEVs express peripheral node addressin (PNAd), and the interaction between L-selectin and PNAd mediates the localization of naïve lymphocytes in NALT,^113^ allowing NALT to be replenished by lymphocytes via HEVs. The B-cell zone contains IgD and IgM B cells, and the T-cell-enriched zone contains CD4 + Th0 cells, CD8αβ+ and CD8γδ + T cells, with CD4 + T cells predominating over CD8 + T cells at steady state.^114^ High densities of dendritic cells (DCs) are present in the nasal cavity of mice. Lee et al. classified them into subpopulations and identified a dense network of CD11c^hi^ cells in NALT with the classical ‘dendritic’ morphology. Upon antigen exposure, these cells lose their dendrites and migrate deeper into the nasal tissue.^115^ Similarly, DCs are extensively present in the human nasal mucosa,^116^ encompassing plasmacytoid dendritic cells (pDCs) and myeloid/conventional dendritic cells (cDCs).^117^ pDCs play an crucial role in sensing and responding to viral infections by rapidly producing large amounts of type I and type III interferons and secreting cytokines, while cDCs activate T-cells through antigen presentation.^118^ Notably, non-plasmacytoid dendritic cells can recognize double-stranded RNA via protein kinase R and secrete high levels of type I interferon in response to viral infection.^119^

The mucosal immune system is crucial for host defense against pathogen invasion. Functionally, the nasal mucosal immune system is divided into induction and effector sites and he link between them occurs mainly through lymphocyte homing. NALT is identified as one significant induction site for mucosal immunity^120^ and serves as the initial lymphoepithelial barrier against respiratory viruses. SARS-CoV-2, a cytopathic virus, induces focal host cell death and cytokine release, which further activate immune cells.^121^ The recruitment and maturation of DCs following viral infection appear to depend on this process.^122^ Concurrently, damage-associated molecular patterns (DAMPs) and viral structures are recognized by the pattern recognition receptors (PRRs) of innate immune cells, triggering intracellular pro-inflammatory and antiviral responses. For instance, during the early stages of viral infection, type I and type III IFNs are co-produced by pDCs and cDCs.^123^ In addition, DCs are the primary APCs at the mucosal barrier in mammals. They can take up antigens by directly or indirectly absorbing substances released by M cells, which in turn activate T and B cells to initiate adaptive immunity.^115,124^ Th0 cells, upon exposure to different antigens, differentiate into various Th subpopulations, including Th1, Th2 and Th17.^33^ The activation of T cells further promotes the formation of GC in the NALT, where B cells undergo IgA class switching and affinity maturation, forming virus-specific antibody-forming cells (AFCs) and memory B cells with high-affinity IgA.^125^ Then, antigen-specific CD4 + T cells and AFCs generated in NALT migrate to effector sites, such as the respiratory mucosal lamina propria and intraepithelial lymphocytes.^27^ Eventually virus-specific secretory IgA (dimeric IgA) is secreted into the respiratory tract via the mucosal epithelium, where it binds to the glycoproteins on the viral surface, neutralizing the virus. Recent research indicates that secretory IgA confers protective effects for at least 8 months after SARS-CoV-2 infection, indicating durable mucosal immunity.^126^ Although studies on d-NALT in mice are limited, current research highlights its critical role in viral infection protection. Following viral infection, a significant presence of AFCs and the persistence of IgA-producing cells in d-NALT^127^ lead to a more sustained antibody response compared to o-NALT.^128^

In addition to limiting further invasion of respiratory viruses, intranasal immunization induces the establishment of protective lung immunity by stimulating IgA-secreting, locally resident B-cell populations.^129^ The effectiveness of current intranasal vaccines relies on NALT,^115^ which, upon proper antigenic stimulation, typically elicits effective humoral and cellular immune responses at both mucosal and systemic levels.^130^ Furthermore, intranasal immunity promotes IgA secretion from distant mucosal sites due to the connection of the common mucosal immune system.^131^ However, mucosal barriers limit the effectiveness of intranasal vaccines, posing a significant challenge in vaccine development.^132^ Fu et al. developed a self-healing hydrogel subunit vaccine and its efficacy in delivering antigens through nasal barriers and enhancing systemic and mucosal immunity.^133^ In addition, Zhang et al. improved non-viral vectors for intranasal DNA vaccines, resulting in stronger immune responses.^134^ Relevant aspects of intranasal vaccines will be discussed subsequently.

### Characteristics of lowerer respiratory tract mucosal immunity

#### Structure and mucosal immune microenvironment of the lower respiratory tract

**Fig. 3:**
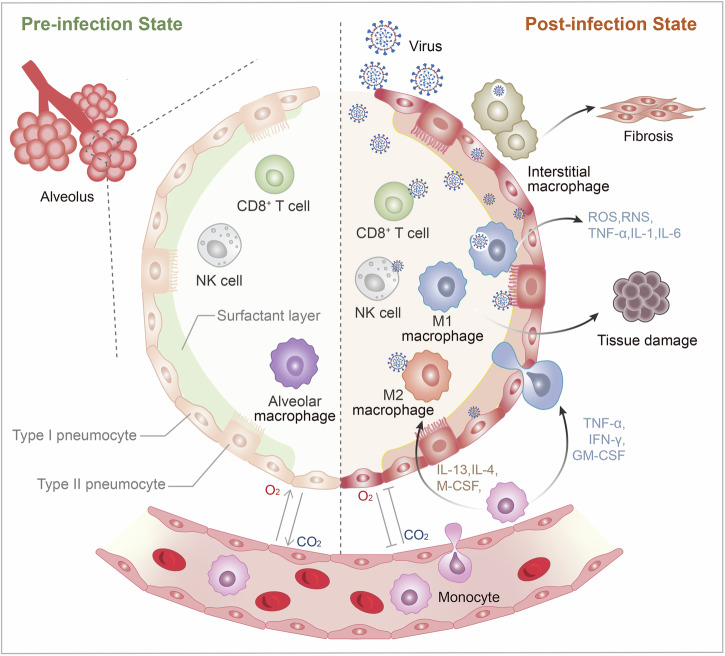
Mucosal immune system in the lower respiratory tract. Type II alveolar epithelial cells are susceptible to severe acute respiratory syndrome coronavirus-2 (SARS-CoV-2) infection. After infection, the secretion of pulmonary surfactant is inhibited, the composition of alveolar surface mucosa changes, and gas exchange is blocked. Monocytes in the blood vessels are the source of alveolar macrophages. Most alveolar macrophages are differentiated into the M1 type after being infected by SARS-CoV-2. SARS-CoV-2 engulfed by M1 type macrophages is more likely to get leaked and cause more serious infection. Additionally, macrophages in the pulmonary interstitium become activated after endocytosis of SARS-CoV-2, resulting in pulmonary fibrosis. Antibody-dependent enhancement can lead to more severe infections. SARS-CoV-2 spike protein binds to neutralizing antibodies, and the Fc segment of the antibodies easily binds to CD16 on the surface of the macrophages, enabling the macrophages to swallow SARS-CoV-2 and cause infection. In infected macrophages, inflammasomes form and initiate pyroptosis, while releasing cytokines and chemokines to trigger cytokine storms

Even if the composition and function of LRT are roughly consistent with URT, differences still exist (Fig. 3). The principal passages of LRT consist of the trachea, bronchi and bronchioles. Within the lung tissue, each bronchus subdivides into secondary and tertiary bronchi, which continue to bifurcate into smaller airways known as bronchioles eventually leading to alveoli. The trachea, bronchi, and bronchioles serve as conduits and transport air from the external environment into the lung, and airborne pathogens and particles including coronavirus, also enter the airways. As a physical barrier, the mucus and airway epithelial cells help to filter a majority of foreign substances out. The mucus mainly produced by goblet cells can absorb pathogens and suppress their transmission; then enough mucus accumulates to form sputum, and ciliated epithelial cells move the sputum upward via their cilia and expectorate it.^135^ Surfactants are complexes comprising unique phospholipids and proteins, with hydrophilic and hydrophobic domains: the hydrophilic head in the membranes and hydrophobic tails in the air, which moisten the surface and reduce surface tension at the air-liquid interface of the airways.^136^ Stromal cells and haematopoietic cells constitute the cellular scaffold of LRT, and alveolar epithelial type I and II cells (AT I/II cells) are the dominant composition of stromal cells in the lung,^137^ among which AT I cells cover 95% of the alveolar epithelium and AT II cells account for the remaining.^138^ AT I cells contribute to the gas exchange at the blood-air barrier, and AT II cells produce pulmonary surfactant (PS) to regulate alveolar surface tension and prevent alveolar collapse during exhalation by reducing elastic recoil.^139^ Covering the surface of the alveoli, PS also has a protecting function, but pathological events like coronavirus intrusion enable the breakdown of PS. AT II cells are susceptible to coronavirus because of their high expression of ACE2,^140,141^ and the infection of AT II cells largely reshapes the immune microenvironment and decides the clinical pulmonary symptoms.^142^

Alveolar macrophages (AMs) will be recruited during infection. There are two AM phenotypes: the proinflammatory M1 and anti-inflammatory M2 AMs. Cytokines (TNF-α and IFN-γ) from activated Th1 cell responses promote M1 AM polarization. As a result, excessive inflammatory cytokines grow up into the local cytokine storm and induce acute lung injury.^143^ Furthermore, lung injury sustains the cascade amplification of inflammatory effect and leads to systemic inflammation.^144^ Moreover, IFN-γ activates aryl hydrocarbon receptor (AhR) on AT II cells, leading to upregulation of the expression of ACE2 and mucins.^145,146^ Consequently, the infection expands, and redundant mucins deposit and gradually impair the exchange of O_2_ and CO_2_. AMs can degrade coronavirus in lysosomes and limit its spread. Nevertheless, compared with M2 AMs, M1 AMs have more acidic endosomes that are able to phagocytose S protein during the transport passage to lysosomes,^147^ and the activated cathepsin L in acidic endosomes enhances the cleavage of S protein,^148^ thus favoring membrane fusion and facilitating the entry of coronavirus RNA from the endosomes into the cytoplasm, where RNA achieves replication and packages into virus particles for release. Therefore, M1 AM polarization leads to more serious symptoms. With respect to the pulmonary interstitium, clinical pathological symptoms such as pulmonary fibrosis and pulmonary interstitial edema are frequently reported.^149–151^ Extracellular matrix (ECM) deposition is a characteristic feature of lung fibrosis induced by the abnormal proliferation of fibroblasts. A newly identified profibrotic phenotype of monocyte-derived CD163+ macrophages can accumulate in the pulmonary interstitium and alveoli and interact with fibroblasts to be involved in potent profibrotic pathways.^152^ In addition, the alternative entry receptor CD147 is verified to be associated with pulmonary fibrosis.^153^

Human influenza virus infection is the most common in the respiratory tract. The virus invades the respiratory epithelium via cleaved hemagglutinin.^154^ The soft palate is the major source of viral transmission because it is enriched in α 2,3 and 2,6 sialic acids that conduce to hemagglutinin-dependent infection.^155^ For infected individuals, the severity of the associated disease depends significantly on the extent to which the virus invades the lower respiratory tract. In particular, the infection of alveolar epithelial cells disrupts the essential gas exchange and facilitates viral exposure to endothelial cells. Upon breaching this delicate layer, exposure of cytokines and viral antigens to the endothelial layer can enhance inflammation.^156^ Endothelial cells become a significant source of pro-inflammatory cytokines, influencing the intensity and nature of subsequent innate and adaptive immune responses.^157^ Ultimately, the compromised ability of the lung to fulfill gas exchange can arise from various non-exclusive mechanisms including airway obstruction, disruption of alveolar structure, loss of lung epithelial integrity due to direct epithelial cell killing, and degradation of the essential extracellular matrix responsible for maintaining lung structure.^158^

As to bacteria, *Mycobacterium tuberculosis* is the causative agent of tuberculosis (TB) that is a primary contributor to deaths in infectious diseases. Humans are the exclusive known natural host and reservoir of *M. tuberculosis*, and DNA evidence suggests that *M. tuberculosis* has undergone co-evolved with Homo sapiens.^159^ *M. tuberculosis* primarily resides within and among innate immune cells, macrophages in particular. Typically, pathogens are eliminated through the fusion of phagosomes with lysosomes, resulting in the acidification of the pathogen-containing phagolysosome.^160^ However, *M. tuberculosis* has employed many strategies to inhibit phagosomal maturation and phagolysosomal generation in order to survive and replicate in macrophages. Phthiocerol dimycocerosates, lipids from *M. tuberculosis*, can mediate escape from the phagosome and host death.^161^ Also, a glycosylated *M. tuberculosis* phosphatidylinositol prevents phagolysosome biosynthesis to escape killing.^162^ What’s more, secretes several enzymes like phosphatases SapM and PtpA or serine/threonine kinases PknG to interfere with phagosomal maturation.^163–165^

#### Role of secreted neutralizing antibodies in lower respiratory tract immunity

With the help of Th2 cells and a series of stimulus signals, naïve B cells sleepy in GC matching the specific antigen to its B cell receptor (BCR) are activated for proliferation and differentiation.^166^ Differentiated plasma cells continuously produce antibodies, and the level and occurrence of neutralizing antibodies are bound up with the severity of the disease.^167,168^ Further, neutralizing antibody responses last at least 5 months after infection, which may lower the chance of reinfection.^169^

The mucosal antibodies, most of whom are neutralizing antibodies and persist long via epithelial cells arrive at the mucosa surface. On their basolateral surface, epithelial cells express polymeric Ig receptors (pIgR) that link with secretory antibodies, endocytose in vesicles, upward transport, cleave, and release them into the mucus.^170^ Once coronavirus attacks the host, IgM, a common mucosal antibody, is the first generated isotype against the novel antigen. Within a week, the predominant antibody, IgA in the mucosal immune system is detectable.^171,172^ The conversion in antibody isotype, owing to the variable splicing of transcripts in mature B cells, is termed class switch, which does not alter the variable region of antibodies but changes the heavy chain constant region.^173^ The change in the constant region determines whether an antibody can be transcytosed through the epithelial layer at mucosal surfaces,^174^ which explains why IgM and IgA instead of IgG are the main mucosal antibodies. Secretory IgA (SIgA) and SIgM are homopolymers linked with the J chain. Usually, SIgA is dimeric and SIgM is pentameric.^175^ Dimeric SIgA, the primary form of mucosal SIgA, is 15 times more potent than IgA monomer referring to the neutralizing effect.^176^In parallel, pentameric IgM surpasses monomeric IgM in potency by approximately 96-fold.^177^ Hence, polymerism prominently augments the antiviral activity of IgA and IgM. They are transported by epithelial cells to the mucosa to neutralize coronavirus. SIgA and SIgM interfere with the earliest steps in the infection process by blocking pathogens from adhering to the airway epithelium and directly neutralizing them, and the anti-spike SIgA is more stable than serum IgA.^178,179^ Previous studies have shown that IgG positivity may be transient or absent in addition to IgA positivity in mild or asymptomatic infections.^180^ In a serology test in Germany, IgA positivity was identified in IgG-negative individuals without a known history of COVID-19.^181^ Moreover, when only mucosal immune responses occur (early stage of infection), mucosal SIgA was observed in cases without detectable serum levels of IgA and IgG.^182^ These findings indicate that SIgA is a reliable and stable biomarker to diagnose COVID-19, and it is easy to collect in saliva. Except for the respiratory tract and saliva, anti- coronavirus SIgA also appears in tears, breast milk and stool,^183–185^ which proves the cubicity and completeness of mucosal humoral immunity. In early coronavirus -specific antibody response, mucosal homing IgA plasmablasts expand, and IgA plays a dominant role in early neutralizing antibody response.^186^ Ejemel M. et al. characterized a human-derived monoclonal IgA, MAb362, which overlapped with the ACE2 structural binding epitope on the S protein, to neutralize coronavirus. Moreover, SIgA shows robust immune memory; after receiving Moderna or Pfizer-BioNTech COVID-19 (BNT162b2) mRNA vaccines, higher levels of SIgA are inducted in participants with prior infection compared to individuals without pre-exposure to coronavirus.^187^ Since a short maintenance time, IgM does not gain too much attention. However, persistently unconventional IgM-specific responses have been reported in both infection and vaccination cases,^188,189^ which suggests a failure to eliminate viruses completely in a short time or a reflection of reinfection.

With more widespread variants exhibiting harsher virulence and wider transmission, the resistance to antibodies and antibody evasion of SARS-CoV-2 variants and subvariants have attracted increasing attention.^190,191^ The neutralizing immunity against wild-type (WT) SARS-CoV-2 decreases across variants, regardless of how it is acquired, by direct infection, or vaccination.^190,192–194^ Therefore, the search for broad-spectrum neutralizing antibodies is important to cope with known and emerging variants. The WT coronavirus spike-specific mucosal IgA also offers protection against SARS-CoV-2 Omicron variant, suggesting that mucosal IgA has broad potency against SARS-CoV-2 variants.^195^ Similarly, nasal delivery of engineered IgM can reduce the resistance and improve the efficacy of immune response against the three variants,^196^ a phenomenon also occurring in human-derived IgM.^197^ Both studies mentioned that cloned identical IgG does not have similar efficacy as IgM; however, the reason for this is still unknown. Published studies that isolated broad neutralizing antibodies (bnAbs) from the total mAbs found that the proportion of identical heavy chains is high in bnAbs. In IgG screening, RBD-targeting bnAbs prefer the heavy chain germlines VH3-53, VH3-66, and VH1-69,^198–200^ which suggests the relevance of heavy chains with bnAbs. Another antibody study has reported that similar encoded motifs on heavy chain germline VH1-2 favor recognizing specific residues on RBD.^201^ Therefore, studies on the conformation of SIgA and SIgM heavy chains interaction with the S protein are valuable but lacking. In addition to various heavy chain genes, the influence of the J chain on heavy chain conformation is worth considering.^175^ The factors affecting the immune escape of coronavirus to antibodies are not limited to the heavy chain. Targeting different binding epitopes of the virus determines the distinct mechanisms of the neutralizing antibodies. The known epitopes of the S protein encompass the N-terminal domain and RBD within the S1 subunit, and within S2 subunit, epitopes targeting the stem helix and fusion peptide regions are identified.^202,203^ If the targeted epitopes are conserved (such as the S2 domain and N-terminal domain of the S1 domain) in variants or even in other coronaviruses, these induced antibodies can provide extensive protection.^204,205^

#### Structure and immune microenvironment of bronchus-associated lymphoid tissue (BALT)

Induced bronchial-associated lymphoid tissue (iBALT), one of tertiary lymphoid structures (TLS) in the lungs, usually forms around the bronchi and in the perivascular space in response to infection or inflammatory stimuli.^206^ iBALT is typically characterized by the B220 + B-cell follicles and a supporting network of CD35 + CXCL13+ follicular dendritic cells (FDCs), and has the capacity of generating germinal center (GC) responses. However, in Pseudomonas aeruginosa-treated mice, atypical B-cell follicles have been identified, featuring podoplanin (PDPN) + CXCL12+ fibroblast-like stromal cells in place of FDCs.^207^ T-cell compartments, containing CD4 + T-cells, CD8 + T-cells along with CD11c+ DCs, are located around the B-cell follicles. The presence of DCs is crucial for the maintenance of iBALT following viral infection.^208^ In addition, CD4 + T cells have been identified within B-cell follicles.^209^ For instance, T follicular helper (Tfh) cells in the GC drive the affinity maturation and further differentiation of antigen-specific B cells through CD40L and IL-21 expression.^210^ The high expression of CXCR5 facilitates the migration of Tfh cells to B-cell follicles in response to CXCL13. Recent studies have extensively revealed the heterogeneity of Tfh cells. In secondary lymphoid organs (SLOs), there exist T follicle-regulating (Tfr) cells which control the amplitude of the GC responses and natural killer T cells (NKT, NKTfh) which boost B cell priming.^211,212^ Additionally, a new subpopulation of Tfh cells, termed T-resident helper cells (Trh), has been observed in TLS, and they promote the formation of B-resident memory (B_RM_) cells and CD8 + T-resident memory (T_RM_) cells maintenance.^213^ In addition to the B-cell and T-cell compartments, iBALT contains CCL21+ PNAd+ high endothelial vesicles (HEVs), typically forming near the periphery of the B-cell follicles and recruiting CCR7-expressing naïve and central memory cells from the bloodstream.^214^ Moreover, similar to SLOs, iBALT features LYVE-1+ Prox-1+ lymphatic vessels (LVs) near the B-cell follicles, although their exact function remains undetermined. SLOs have afferent LVs that deliver antigens and APCs to the lymphoid tissue, and efferent LVs that drain activated lymphocytes.^215^ Given that iBALT is characterized by the local presence of antigens, the necessity for LVs to transport antigens and APCs to iBALT warrants further investigation. Notably, LVs express the chemokine CCL21 and may perform the similar function to that of HEVs.^215^ LVs can attenuate the inflammatory response through fluid drainage. Impairment of the drainage function of LVs leads to the persistent presence of antigens and immune cells at the injury site, facilitating the formation and maintenance of TLS.^216^

Infection with SARS-CoV-2 in young children is less severe than in adults,^217^ as children are capable of mounting an effective immune response to respiratory pathogens. A recent study found that BALT is abundant in the lungs early in life, promoting local immunity against multiple pathogen challenges. However, it becomes increasingly difficult to form with age.^218^ This finding may explain the observed differences in disease severity between children and adults. Typically, the formation of iBALT follows 3 major steps:^219^ (1) stromal activation, (2) immune cell recruitment, and (3) maturation and maintenance. The highly ordered compartmentalization of T-cells, B-cells, and myeloid cells in the SLOs is essential for generating of the effective immune response. Stromal cells are responsible for the formation and maintenance of this compartmentalization^220^ and are highly heterogeneous.^221^ Similarly, the maintenance of the function of iBALT requires a network of specialized stromal cells. Fibroblasts are the predominant non-hematopoietic stromal cells, and the formation of initial TLS necessitates the reprogramming of resident fibroblasts in non-immune organs to acquire the immunofibroblast phenotype. This process involves three phases: priming, expansion and maturation.^222^ Primed fibroblasts upregulate the expression of various adhesion molecules, facilitating interactions between stromal cells and lymphocytes. Fibroblast priming is followed by an active phase of proliferation. Mature immunofibroblasts ultimately secrete a variety of chemokines including CXCL13, CCL19, and CCL21, and this process is regulated by lymphocytes and LTβR signaling. The chemokine-rich niche facilitates immune cell recruitment. Additionally, the recruitment of numerous immune cells requires a phenotypic shift in CD31+ MadCAM- PNAd- endothelial cells (ECs) to CD31+ MadCAM+ PNAd- immature HEVs and CD31+ MadCAM- PNAd+ mature HEVs, dependent on TNFR and LTβR signaling.^223^ TNFR signaling aids the formation of immature HEVs, while LTBR signaling promotes the formation of mature HEVs. Finally, the segregation of B and T cells in iBALT is driven by the development of the stromal network. Fibroblasts differentiate into FDCs, which secrete CXCL13 and B cell activating factor (BAFF), crucial for Tfh cell recruitment as well as B cell migration and survival.^223^ Differentiation of T-zone reticular cells (TRCs) supports the recruitment of CCR7 + T cells and DCs through the secretion of CCL19 and CCL21.^224^ In addition, in the SLOs, there exist marginal reticular cells (MRCs) responsible for antigen capture and delivery and CXCL12-expressing reticular cells (CRCs) required for the recruitment of CXCR4 centroblasts and an efficient GC response.^225^ By contrast, the mature stromal fibroblast subpopulation in TLS has not been well characterized.

Prolonged exposure to antigenic environments stimulates the formation of iBALT. However, iBALT can persist in the lungs for several months after antigens are cleared, which relies on cytokines and intercellular interactions.^226^ Established iBALT functions similarly to SLOs by recruiting naïve B and T cells and supporting their response to unrelated antigens. This enhances the host immune response to respiratory viruses and facilitates more effective viral clearance.^227^ iBALT is crucial for maintaining immune memory.^228^ Additionally, CD4 + T_RM_ cells in lung tissue have been reported to provide optimal protection against respiratory viral infections^229^ and the survival of CD4 + T_RM_ cells is dependent on IL-7 production by lymphatic endothelial cells. In addition, the reactivation of CD8 + T_RM_ cells in the lungs is not strictly limited to the type of APCs, and reactivated cells acquire the circulating memory T cell properties and exhibit the accelerated protective response.^230^ It is noteworthy that iBALT formation can lead to protective or pathological outcomes, thereby influencing disease progression.^222^ These outcomes may be influenced by variations in antigenic properties, the duration of antigen exposure, and cytokine signaling.

Intranasal vaccination against SARS-CoV-2 induces iBALT formation in the lungs of mice, but it seems that the absence of iBALT does not diminish vaccine efficacy.^231^ This may be attributed to the relatively delayed initiation of an immune response by iBALT, which is overshadowed by the rapid and robust response from conventional lymphoid organs.^209^ Furthermore, although TLR9 agonists were previously considered to enhance vaccine efficacy as adjuvants, Do et al. found that TLR9 agonists did not improve the antigen-specific CD8 T-cell response induced by intranasal vaccination with the MVA-SARS-2-S vaccine. Instead, they inhibited iBALT formation,^232^ the exact mechanism of which needs further investigation. Intratracheal (IT) administration also promoted the development of iBALT and resulted in higher and more sustained systemic and lung local neutralizing antibody (NAb) titres than intramuscular administration and induced the production of lung T_RM_ cells,^233^ suggesting that IT administration may offer effective protection against viral infection.

### Characteristics of gastrointestinal tract mucosal immunity

#### Structure and mucosal immune microenvironment of Gut-Associated Lymphoid Tissue (GALT)

**Fig. 4:**
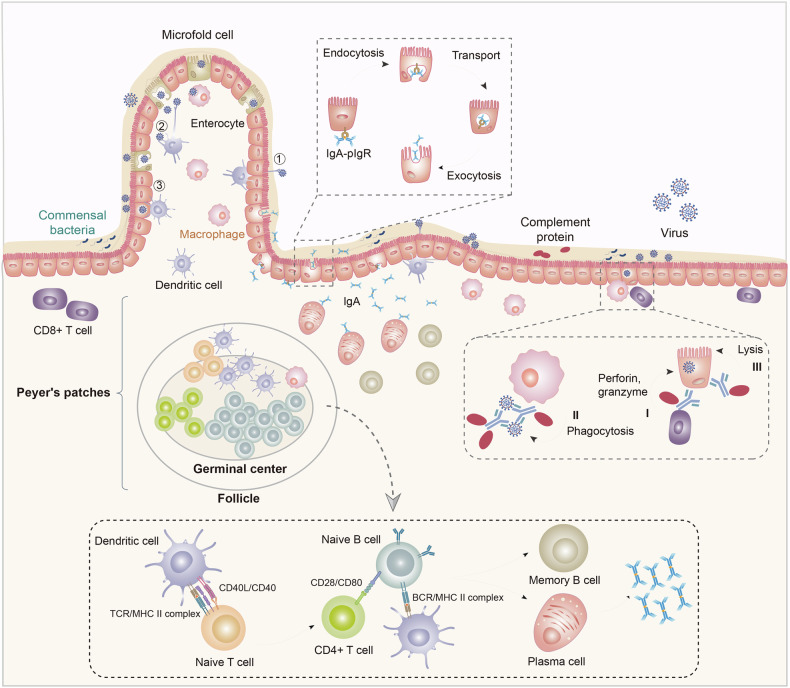
Mucosal immune system in the gastrointestinal tract. Macrophages and dendritic cells below the intestinal epithelial layer are specialized antigen-presenting cells (APC). DCs ingest antigen mainly in three ways: ① DCs extend directly out of the intestinal epithelial cell layer to capture the severe acute respiratory syndrome-coronavirus-2; ② M cells in the epithelial cell layer transport external viruses to the PPs to be ingested by DCs; ③ DCs indirectly ingest coronavirus disease 2019 antigen by ingesting infected intestinal epithelial cells. After presenting antigens, the APC moves down the germinal center of the PPs and lymph follicles and activates naive T and B cells. B cells then differentiate into plasma cells to secrete antibodies. Secretory dimeric immunoglobulin A (IgA) are joined by the J chain to polymeric Ig receptor (pIgR) located only at the basement of the enterocyte. Thereafter, enterocytes endocytose the IgA–pIgR complex, transporting it to the upper side. Finally, pIgR is cleaved and IgA is exocytosed (SIgM can be transported to the mucosa by this mechanism). DCs bind to T-cell receptors on the surface of naive T cells (whereas CD40L on the surface of the T cells binds to CD40 on the surface of the dendritic cells), causing them to differentiate into CD4 + T cells. CD28 on CD4 + T cell and antigen presenting MHC on DCs bind to cell surface receptors of naive B cells, causing them to differentiate into plasma cells and secrete antibodies. Fc domain-mediated functions: The infected cells can express antigens to help antibodies find them. (I) antibody-dependent cellular cytotoxicity, CD8 + T cells recognize infected cells by Fc domain of antibodies, then secret perforin and granzyme to lyse them; (II) antibody-dependent phagocytosis, macrophages (or other APCs) phagocytose antigen-antibody complex and infected cells after binding with the antibodies on them; (III) antibody-mediated complement-dependent cytotoxicity, combining with antibodies, complement proteins form membrane attack complex on the surface of infected cells, which induced cell lysis soon

MALT is abundant mostly in the intestine, such as in the gut-associated lymphoid tissue (Fig. 4). Coronavirus enters the gastrointestinal tract via the oral cavity.^234^ Mesenteric lymph nodes are typical of organized mucosal lymphoid tissues that lie within mesenteric layers. Blood and other lymphoid tissues are connected through high endothelial venules and lymph vessels to ensure lymphocyte migration.^235^ Peyer’s patches (PPs), observable as elongated thickenings of the intestinal mucosa, are specially organized mucosal lymphoid tissues in the gut, except for the mesenteric lymph nodes. The structure and composition of PPs resemble adenoids, and here we focus on the process of antigen-presenting. Owing to the presence of B cells, PPs are regarded as the main inductive sites for gut antibody response.^236,237^

M cells are one of the enterocytes of PPs, which serve as one of the predominant ways APCs interact with antigens (Fig. 4). They endocytose potential antigens like proteins, bacteria, viruses, and non-infectious particles from the apical membrane, and transfer them to the basolateral surface where APCs are rich.^238–243^ Horvath D. et al. proposed a novel intranasal vaccine targeted at mucosal M cells by fusing bacteria-driven Claudin-4 ligand to receptor binding domain,^244^ increasing the immunogenicity of the vaccine and eliciting strong activation of DCs and robust CD4+ and CD8 + T-cells. The mucosal immune system in the gastrointestinal tract involves specialized APCs such as macrophages and DCs located below the intestinal epithelial layer. DCs are leading APCs located beneath the epithelial layer of PPs, broadly classified as classical DCs (cDCs), plasmacytoid DCs (pDCs) and Langerhans cells (LCs); cDCs are in the mucosa and lamina propria, pDCs are in the peripheral blood, and Langerhans cells are in the mucosa and skin.^245,246^ DCs capture antigens through three primary mechanisms: (a) extending directly out of the intestinal epithelial cell layer to capture pathogens; (b) receiving viruses transported by M cells in the epithelial layer to PPs where they are ingested by DCs; and (c) indirectly ingesting virus antigens by consuming infected intestinal epithelial cells. Once DCs present the antigens, they migrate to the germinal center of PPs and lymph follicles to activate naive T and B cells. B cells subsequently differentiate into plasma cells to produce antibodies.

In addition to classical APCs, the mucosal immune microenvironment of the gut also involves various subsets of innate lymphoid cells (ILCs), which play crucial roles in maintaining immune homeostasis and responding to infections.^247^ Distinct subsets, including ILC1s, ILC2s, and ILC3s, are strategically positioned within the gut mucosa, each executing specialized functions. For example, ILC3s are crucial in preserving the integrity of the intestinal barrier through the secretion of IL-22, a cytokine that drives epithelial cell repair and fortifies mucosal defenses against pathogens.^248^ Meanwhile, ILC1s produce interferon-gamma (IFN-γ), which is essential for combating intracellular pathogens,^249^ whereas ILC2s modulate responses to helminths and allergens by releasing cytokines such as IL-5 and IL-13.^250^ Moreover, the impact of ILCs extends beyond the gut, highlighting the intricate relationship of the gut-lung axis.^251^ This axis encapsulates the bidirectional communication between the immune systems of the gut and lungs, where signals originating in the gut can influence lung immunity, and vice versa. ILC2s, in particular, emerge as key regulators within this axis due to their cytokine production.^252^ The same IL-5 and IL-13 produced by ILC2s, which are vital for regulating gut immunity, also play indispensable roles in lung inflammation and defense against respiratory pathogens and allergens.^253^ Consequently, the presence and function of these cells underscore the intricate and dynamic nature of the mucosal immune system, where adaptive and innate immune components synergistically operate across diverse mucosal sites to ensure host protection.

Secretory dimeric IgA is joined by the J chain to the polymeric Ig receptor (pIgR) located at the basement of enterocytes. Enterocytes then endocytose the IgA–pIgR complex, transport it to the apical side, cleave pIgR, and exocytose IgA. Secretory IgM (SIgM) can also be transported to the mucosa via this mechanism (Fig. 4). DCs bind to T-cell receptors on naive T cells, with CD40L on T cells binding to CD40 on DCs, causing differentiation into CD4 + T cells. CD28 on CD4 + T cells and antigen-presenting MHC on DCs bind to receptors on naive B cells, prompting them to differentiate into plasma cells and secrete antibodies. The Fc domain-mediated functions include: (I) antibody-dependent cellular cytotoxicity (ADCC), where CD8 + T cells recognize infected cells via the Fc domain of antibodies and secrete perforin and granzyme to lyse them; (II) antibody-dependent phagocytosis (ADP), where macrophages or other APCs phagocytose antigen-antibody complexes and infected cells after binding to the antibodies; and (III) antibody-mediated CDC, where complement proteins form a membrane attack complex (MAC) on the surface of infected cells in conjunction with antibodies, leading to cell lysis (Fig. 4). All of the DCs are involved in innate immunity in addition to presenting antigens in adaptive immunity. cDCs are the principal source of proinflammatory chemokines critical for recruiting various inflammatory cells,^118^ and pDCs secrete interferon-alpha (IFN-α) against SARS-CoV-2 invasion,^254,255^ both of which can join blood circulation. Studies on the association between COVID-19 and pDCs have verified the deficiency and dysfunctionality of pDCs during COVID-19 and even post-COVID-19;^256–258^ however, there are huge blanks about the relationship between cDCs and COVID-19 need to be filled. cDCs accumulate in the lungs of patients with COVID-19.^259^ LCs lie in the ceiling of epithelium, and their extended dendrites form a continuous network to perceive and collect foreign antigens.^260^ They are independent of blood circulation in the steady state, and circulating monocytes will repopulate LCs during inflammation.^261^ To present antigens, DCs can cross the epithelial layer and directly capture exogenous antigens, whereas M cells transfer antigens to DCs. In addition, DCs can indirectly capture antigens by digesting infected epithelial cells.^262^ DCs possibly present coronavirus indirectly and activate B and T cells in lymphoid follicles, which are the main components of PPs. Heparan sulfate proteoglycans are surface receptors attached by coronavirus, which help DCs to capture the virus.^263^ What’s more, the codeletion of cDCs and LCs inducts weak humoral immune responses after the coronavirus vaccination.^264^ The germane relationship between DCs and coronavirus needs more exploration.

The gastrointestinal tract is the first site of invasion for many viruses into the body; like polioviruses, they enter the gastrointestinal tract through the fecal-oral transmission route, infect intestinal epithelial cells, cross the intestinal mucosal barrier, and ultimately invade spinal nerve cells leading to polio. Fortunately, polio has been under control with the widespread oral poliovirus vaccine, which elicits robust specific mucosal and serum antibody reactions.^265^ Apart from viruses, pathogenic bacteria like *Helicobacter pylori* are also a concern for public health. Adapting to the extremely acidic environment, *H.pylori* usually colonizes in the stomach and duodenum. It will develop gastric and duodenal ulcers or even gastric MALT lymphoma or gastric carcinoma during long-term infection. After infection, epidermal growth factor receptors (EGFRs) of gastric epithelial cells are abnormally phosphorylated through CagA oncoprotein encoding by *H.pylori* or heparin from the host.^266,267^ EGFR pathway activation leads to the high expression of NF-κB and IL-1β, thus DNA damage and autophagy of gastric epithelial cells arising which is considered as early steps for gastric carcinogenesis.^266,268,269^ Meanwhile, the host immune defense like antimicrobial β-defensins also switches on following the activation of EGFR.^270,271^

#### The role of non-neutralizing antibodies and antibody-dependent enhancement

Non-neutralizing antibodies (NnAbs) are the other immunoglobulins that can also recognize antigens and attach to antigens or infected cells, but they cannot neutralize the antigens or prevent their transmission.^272^ Notwithstanding, the role of NnAbs should not be underestimated. More like a positioner, NnAbs tell the immune system where these dangerous invaders are rather than directly eliminate them. Thus we concentrate on the conserved Fc domain of the immunoglobulin that mediates ADCC, ADP, and antibody-mediated CDC. For other viruses, these mechanisms also widely exist.^273–277^ Although NnAbs do not directly neutralize or prevent viral transmission, they play a crucial role in the immune response through several mechanisms. They facilitate ADCC, where they bind to viral antigens on infected cells and engage Fc receptors on NK cells and other immune effector cells, leading to the destruction of these infected cells.^278^ In addition, NnAbs enhance ADP by opsonising viral particles, which are then engulfed and destroyed by phagocytes such as macrophages and neutrophils. They also mediate CDC by activating the complement system, leading to the formation of the MAC, which lyses infected cells. However, NnAbs can also contribute to antibody-dependent enhancement (ADE), where suboptimal antibody binding facilitates increased viral entry into host cells via Fc or complement receptors, potentially exacerbating infection.^279^ In the GALT, these antibodies play a critical role in recognizing and eliminating pathogens, thereby maintaining gut health and preventing the systemic spread of infection.^280^

These Fc effector functions are proven to contribute to COVID-19 control and maintain a longer period compared to neutralization activity.^281^ The Fc γ receptors IIIa (FcγRIIIa/CD16) are exposed on the surface of ADCC effector cells like NK cells, monocytes, macrophages and neutrophils, among which NK cells are proposed to be the major contributors to ADCC in vivo.^282^ The interaction between FcγRIIIa and Fc domain activates the Ca^2+^-dependent signaling pathway and phosphorylates the tyrosine-based activation motif; so that NK cells start to secret cytotoxic perforins and granzymes, killing the infected cells.^283,284^ RBD of the S1 subunit, S2 subunit and N protein are able to elicit ADCC responses,^284,285^ and for BNT162b2 vaccine recipients, Fc-mediated effector functions with weak neutralizing activity were detected after a single dose,^286^ and strong CD107a NK cell expression accompanying agitated ADCC was observed after the second vaccination.^284^

Phagocytosis is a crucial immune defense mechanism in that phagocytes engulf pathogens and infected cells. In the beginning, IgG and IgM dispose and mark these targets which is called opsonization; then mainly under the aid of FcγRIa (CD64) and FcγRIIa (CD32), as well as FcγRIIIa, phagocytes including macrophages, monocytes, DCs and neutrophils engulf opsonized pathogen particles.^287,288^ As to IgA-mediated phagocytosis, FcαRI (CD89) plays a momentous role.^289,290^ ADP is one of the most effective means to remove foreign pathogens and infected cells against the influenza virus, it also occurs in COVID-19 patients attempting to restrain spread.^281^ On the contrary, it is found that APC is possible to initiate ADE worsening patients’ condition, which may explain SARS-CoV-2 destruction during inflammatory responses. The surface of phagocytes did not express enter receptors. Fc domain and FcR acting as the ‘Trojan horse’, provide the opportunity for coronavirus to infect these cells. Non-neutralizing antibodies link SARS-CoV-2 to FcγRIIIa AMs, boosting the ability of AMs to engulf coronavirus.^291^ Likewise, Maemura et al. recurred ADE mediated by FcγRIIa and FcγRIIIa in monocyte-derived macrophages in vitro, but they did not observe an anomalous increase of cytokine and chemokine.^292^ Vitro ADE does not necessarily simulate the authentic vivo situation; it is more biased that ADE induces inflammation in vivo and ADE-mediated IL-6 aberrance is one reason for severe COVID-19.^293^ Following the duplication of coronavirus in AMs, inflammasomes are stimulated, leading to pyroptosis and the release of inflammatory cytokines.^291^ These cytokines including IL-6, TNF-α and IFN-I, disrupt the production of PS by AT II cells and polarize AMs to the M1-like phenotype,^294,295^ and consequent to the disruption of PS production, coronavirus can infect AT II cells and O_2_–CO_2_ exchange is impaired. Normally, most AMs are the M2 phenotype; however, to fight pathogen invasion, M2 AMs must switch to M1 AMs to rapidly trigger inflammatory responses. In addition, antibodies induced by mRNA vaccine also have the potential to elicit ADE in vitro,^296^ whereas a study indicated that BNT162b2 vaccine-elicited IgG are enriched in Fc sialylation and highly fucosylated, which seemed to have lessened inflammatory potential.^297^

The function of CDC has been discussed earlier. Fc domain of IgG and IgM can bind with a subcomponent of C1 complement (C1q), but the affinity is significantly affected by antigen binding to Fab domain;^298,299^ thereby CDC is flexibly regulated depending on the presence of antigens. ABO antibodies are natural and universal that may be regarded as one of NnAbs for coronavirus. Multiple investigations have mentioned that the infection rates and mortality tilted to blood type A and deviated to type O,^300–303^ which suggests discordant influences from different types of preexisting ABO antibodies.

Furthermore, NnAb CV3-13 can enhance SARS-CoV-2 N-terminal binding and synergize with nAbs to delay transmission and protect from lethal infection.^304^ Seeing that the multi-functions of NnAbs, irritating humoral immunity with the Fc domain of NnAbs has already been invested in vaccine and inhibitor development,^305–307^ which will be covered in the fifth section.

#### Interaction between intestinal immune homeostasis and mucosal immunity

Commensal microbiota, which is selected by the host, chronically inhabit all the surfaces of mucous membranes. Normally, the host offers a suitable habitat for commensal microbiota, and the metabolites or substances produced by these commensal bacteria modulate the mucosal immune response. Immunoglobulin A (IgA) is the most abundant antibody isotype in mammals, constituting over 80% of all antibody-secreting plasma cells under steady-state conditions.^308,309^ IgA is particularly enriched on barrier surfaces such as the intestinal mucosa, where it forms the first line of defence along with innate regulators such as mucin and antimicrobial peptides.^310,311^ IgA is known to coat the commensal microbiota residing in the gut and to resist intestinal pathogens. In humans, IgA can be divided into serum and secretory types based on its distribution, with secretory IgA (SIgA) being the predominant mucosal antibody. Most IgA-secreting plasma cells are located in the intestinal mucosa, and SIgA can neutralize pathogens or toxins produced by intestinal bacteria, thereby mediating microbial homeostasis in the gut. Researchers have found that gut homeostatic IgA is a naturally occurring multi-reactive antibody with innate specificity for microbiota. The findings suggest that IgA antibodies, although derived from the adaptive immune system, have similar innate recognition properties that may help to adapt to the large and dynamic exogenous microbiota and dietary antigens encountered at mucosal surfaces.^312^ Bunker et al. discovered that antibodies produced by naïve plasma cells in the small intestine are recirculated and concentrated in Peyer’s patches independently of external antigens and T cell assistance. The resultant polyreactive IgAs are released into the intestinal lumen, where they bind to microbial surface glycans, demonstrating innate recognition of the gut microbiota. Polyreactive IgAs are thought to result from the coevolution of the host and microbiota, contributing to symbiotic homeostasis maintenance. Previous studies have shown that IgA enhances adherence of Escherichia coli, Bifidobacterium lactis, and Lactobacillus rhamnosus to epithelial cells in tissue culture,^313,314^ suggesting that these microorganisms may benefit from IgA in establishing a mucosal bacterial community. It is proposed that under healthy conditions, IgA promotes mucosal colonization by microbiota with beneficial properties, whereas disease states may induce (or be caused by) IgA responses to pathogens or pathobionts that disrupt healthy microbiome balance. Indeed, computational models suggest that IgA plays a dual role in maintaining indigenous mucosal populations and eliminating invasive pathogens.^315^ Naturally polyreactive antibodies constitute the majority of broadly neutralizing antibody responses to influenza virus and HIV,^316–319^ and that these bnAbs are of the same type that drive the homeostatic intestinal IgA response,^312^ demonstrate the protective value of polyreactive antibodies in a variety of homeostatic and pathological contexts; exploiting the naturally polyreactive IgA response may provide opportunities to elicit bnAbs by mucosal vaccination.

Although SARS-CoV-2 predominantly infects the respiratory tract, there is evidence that the gastrointestinal tract is also involved.^7,320^ Because ACE2 and TMPRSS2 are highly expressed in the intestinal epithelium, the gut is sensitive to coronavirus.^7,320^ The gastrointestinal tract, in particular, owns the highest abundance and the largest richness of microbial populations in the human body, which play a marked role in body homeostasis and disease. They assist in the uptake of nutrients and degradation of toxins, impede pathogenic bacteria from gaining living spaces, and inhibit inflammatory reactions conducive to infection by pathogenic bacteria.^321^ Furthermore, they regulate immune cell differentiation and activation, and broaden their impacts on the whole body through the gut-lung, gut-liver and gut-brain axis.^322–324^ The host and commensal bacteria maintain a win–win balance.

However, the function of the commensal microbiota in the gut is usually underestimated. A few studies have confirmed that the gastrointestinal microbiota assists in regulating intestinal immune homeostasis and resisting infection.^251,323^ Hence, gastrointestinal microbiota are potential to prevent the colonization of coronavirus in the gut and, to some extent, inhibit infection. Gastrointestinal microbiota can regulate gene expression in the gut, such as those of the Bacteroides species, which downregulate colonic ACE2 expression and may inhibit coronavirus infection.^325^ Furthermore, four Bacteroides species were found to be negatively correlated with fecal coronavirus load in humans.^326^ In randomized clinical trials, probiotic supplements can regulate immune cells and generate inflammatory chemical messengers to respond to the respiratory tract infection.^327–329^ These studies demonstrate that the gastrointestinal microbiota and respiratory system have a close relationship, suggesting that the gut–lung axis function in COVID-19 should be reconsidered.^330^

SARS-CoV-2 attempts to break the barrier established by gastrointestinal microbiota. Clinical studies have demonstrated that gastrointestinal microbiota homeostasis is disturbed and the diversities of microbiota are significantly reduced.^326,331–334^ Beyond the gastrointestinal microbiota, the gut viral community also distinctly alters according to the gut virome research.^335,336^ Compared with healthy individuals, those patients with COVID-19 have low abundances of *Faecalibacterium*, *Eubacterium*, *Coprococcus*, *Ruminococcus*, *Lachnospira*, and *Roseburia* but high abundances of *Enterococcus*, *Rothia*, and *Lactobacillus.*^337^ Additionally, dysbiosis of gastrointestinal microbiota is associated with disease severity and dysfunctional immune responses.^333,338^ Summarily, the microbiota in patients with COVID-19 is deficient in beneficial commensal bacteria and abundant in opportunistic bacteria that further eventuate cytokine storms and co-infections, making disease symptoms more serious.^339^ The reason for microbiota dysbiosis is still complicated and abstruse, even though several hypotheses have been proposed. But one thing that the alternations from the host gut environment including cell infection, cell-mediated immune reactions and release of special secretions incur the dysbiosis could be admitted.^337,340,341^ Viana S. D. et al. thought ACE2 imbalance was the key factor.^342^ ACE2 originally mitigates the deleterious effects of angiotensin II, but ACE2 shedding off owing to SARS-CoV-2 infection impaired the renin-angiotensin system, thus exacerbating the inflammation.^343^ Besides, Bernard-Raichon L. et al. assumed bacteriemia followed by gut mucosal damage causes gut microbial translocation into the circulatory system.^344^

Moreover, the gastrointestinal microbiota can participate in the regulation of pulmonary inflammation caused by coronavirus through the gut-lung axis. The crosstalk between the respiratory mucosa and the gut microbiota is known as the gut-lung axis. The communication is developed mainly by the transfer of microbial metabolites, translocation of microbiota, and the spread of cytokines.^345^ Many reviews have summarized the linkage between the gut-lung axis and COVID-19; however, there is still a long way to go before applying this concept to the field of translational medicine. Present treatments prefer direct targeting achieving significant results, while the gut-lung axis tends towards indirect regulation and slowly advances, like following a zigzag route.

## Mucosal immunity related diseases

The structure and function of the mucosal immune system have close correlations with its methods of tackling disease, and correspondingly, mucosal-related diseases may utilize its properties to boost themselves. Mucosal immunity is involved in the immune response to viral infections and is also essential in the pathogenesis of inflammatory diseases, autoimmune diseases, and certain cancers. Apart from COVID-19, we summarize clinical symptoms, pathogenesis and mucosal immune response of frequent or high-risk mucosal-related diseases (Table 1),^59,95,160,346–362^ trying to provide a comprehensive landscape of mucosal immunity.

**Table 1:** Indications of mucosal-related diseases

Diseases	Primary localization	Clinical symptoms	Pathogenesis	Mucosal immune response	Refs.
Adenoidal hypertrophy	URT	The enlarged adenoid may cause obstructions of nasal passages and eustachian tubes, persistent congestion, nasal drainage, sinusitis, otitis media;	Chronic infection (e.g., Streptococcus pyogenes) and allergy (e.g., house dust mites), acid reflux elicit lasting inflammation;	Abnormalities in the number and function of various lymphocyte subsets in the adenoid;	37–39
Herpangina	URT	A high fever and blister-like sores in the mouth and throat;	Infection of Coxsackievirus-A, Enterovirus-A and Echovirus;	Serotype-specific antibody response, macrophage proliferation and phagocytosis enhancement;	40,41
Allergic rhinitis	URT	Nasal congestion, clear rhinorrhea, sneezing, postnasal drip, and nasal pruritis;	Common allergens including pollen, mould spores, house dust mites, and flakes of skin or droplets of urine or saliva from certain animals;	Th2 cell-induced IgE response, histamine secretion by mast cells, IL-4 and IL-13 facilitating the infiltration of eosinophils, T-lymphocytes, and basophils;	42,43
Mucous membrane pemphigoid (one of autoimmune blistering disorders)	URT	Red, blistering lesions, ulceration, and subsequent scarring;	Linear deposition of IgG, IgA, or C3 along the epithelial basement membrane zone		44
Seasonal influenza	URT/LRT	Fever, nonproductive cough, headache, muscle and joint pain, sore throat and a runny nose;	Infection of influenza A or B viruses;	Collaborative neutralization reactions of SIgA and IgG in LRT, induction of autonomous memory of alveolar macrophages;	45,46
Respiratory syncytial virus infection	URT/LRT	rhinorrhea, nasal congestion, cough, sneezing, and bronchiolitis or even viral pneumonia;	Infection of respiratory syncytial virus;	IL-17-dominated immune response, neutrophilic inflammation;	47,48
Pulmonary tuberculosis	LRT	Dyspnea, prolonged cough with mucus, pleuritic chest pain, hemoptysis;	Infection of Mycobacterium tuberculosis, macrophage aggregation induced granuloma formation to evade immune clearance;	Alveolar macrophages phagocytosis and production of cytokines such as TNF-α and IFN-γ, T-cell recruitment in granuloma;	49,50
Pneumocystis pneumonia	LRT	Fever, nonproductive cough, dyspnea, and hypoxemia; with diffuse bilateral ground-	Infection of Pneumocystis; jirovecii in immunocompromised	Alveolar macrophage phagocytosis and production of IFN-γ, TNF-α, IL-1, IL-6; and GM-CSF, protective	51,52

		glass opacities on chest imaging;	patients (like HIV patients);	immune response of SIgM;	
Pneumoconiosis	LRT	Long-term cough with mucus, shortness of breath and chest tightness, subpleural honeycombing and fibrosis;	Inhaled airborne dust and fibers induced macrophage dysfunction and interstitial lung disease;	Smaller particles take-up by alveolar macrophages phagocytosis, release of IL-1, TNF-α and lysosomal enzymes, generation of free radicals, and production of extracellular matrix and matrix metalloproteinases by activated fibroblasts;	53
Asthma	LRT	Dyspnea during eat, speak or sleep, wheezing, coughing especially at night or early morning, and chest tightness;	Chronic airway inflammation induced by factors like allergens, infections, obesity, smoking, systemic eosinophilia, leading to airway obstruction and hyperresponsiveness;	Production of thymic stromal lymphopoietin by DCs and injured epithelial cells eliciting Th2-cell type inflammation;	54,55
Staph infections	URT/LRT/GIT	Antibiotic-associated diarrhea, bacteremia, toxic shock syndrome;	Infection of Staphylococcus aureus;	Junctional integrity alteration of enterocyte, infected epithelial cells eliciting Th2-cell immune response;	56,57
Crohn’s disease	GIT	Abdominal pain and cramping typically in the lower right abdomen, diarrhea sometimes bloody, fistulas, weight loss, fatigue, fever and anemia, broad inflammation in the whole gastrointestinal tract;	Inflammatory bowel disease induced by multiple factors including genetic, environment, microbiota, overactive immune system;	Th1-cell immune response, high level of TNF-α and IL-12 induced autoimmune reaction; Th cell (e.g., Th17 and regulatory T cell) development hindrance because of microbiota dysbiosis, SIgA deficiency obstructing anti-inflammation reaction;	58,59
Ulcerative colitis	GIT	Bloody diarrhea, abdominal pain and cramping, weight loss, fatigue, fever and anemia, limited inflammation in the colon and rectum;	Inflammatory bowel disease targeting the colon’s mucosa;	Th2-cell immune response, high level of IL-4 and IL-13, SIgA deficiency obstructing anti-inflammation reaction;	58–60
Helicobacter pylori infection	GIT	Chronic gastritis, peptic ulcers, dull or burning stomach pain (especially when you have an empty stomach)	Infection of Helicobacter pylori;	Th1-cell immune response, generation and activation of Th17 and regulatory T cells;	61
Celiac disease	GIT	Malabsorptio, diarrhea, fatigue, weight loss, bloating, anemia;	Caused by the ingestion of gluten, proteolytically stable gluten peptides crosslinking with transglutaminase 2 to form complex, the complex eliciting Th1 proinflammatory response and specific antibody production, IFN-γ and IL-15 activating cytotoxicity IELs leading to intestinal epithelial injury;		62

Rotavirus infection	GIT	Severe watery diarrhea, vomiting, fever, abdominal pain;	Infection of Rotavirus;	Th1-cell immune response, SIgA contributing to viruses shedding;	63
Cholera	GIT	Severe watery diarrhea, vomiting, thirst, leg cramps, restlessness and irritability;	Infection of Vibrio cholerae;	Increased mucosal-associated lymphocyte homing, protective immune response of SIgA;	64,65

According to statistics, over 50% of pathogens infect the human body through mucosal surfaces. Diseases that pose significant threats to human life, such as AIDS, meningitis, influenza, toxoplasmosis, tuberculosis, diarrhea, gonorrhea, hepatitis, and severe acute respiratory syndrome (SARS), all originate from mucosal surfaces. Immune responses play a crucial role in protecting the host from pathogens and harmful symbiotic bacteria. However, the fact that pathogens can cause diseases indicates that they can at least temporarily overcome the host’s immune defenses, establishing an infection.

### Mucosal immunity in pathogens

Staphylococcus aureus is a common pathogen causing respiratory infections, mainly transmitted through skin, oral, and vaginal mucosa. Its primary virulence factors are staphylococcal enterotoxins (SEs). In the immunotherapy of SEs poisoning, mucosal vaccination and mucosal antibody IgA play important roles. Studies have shown that after oral administration of the STEBVax vaccine, both serum IgG and fecal IgA in piglet models can recognize SEB.

*Streptococcus pneumoniae* is a common colonizer of the upper respiratory tract. In controlled human infection studies, the nasopharyngeal epithelial cells of healthy participants are invaded but do not develop disease. Between 27 and 65% of children and less than 10% of adults carry this bacterium, reflecting a symbiotic relationship between the bacterium and the host.^363,364^ Local spread, inhalation, or entry into the bloodstream can lead to invasive inflammatory diseases.^365^ It is a major pathogen causing otitis media, community-acquired pneumonia, sepsis, and meningitis. To colonize and persist on mucosal surfaces, *S. pneumoniae* relies on various bacterial factors, with its density and duration of colonization being sufficient for its transmission. For instance, *S. pneumoniae* expresses two enzymes: peptidoglycan N-acetylglucosamine deacetylase (PgdA) and resistance attenuator (Adr), which modify its peptidoglycan, making it resistant to lysozyme abundant on the mucosal surfaces of the upper respiratory tract.^366^ The colonization features of *S. pneumoniae* include adhesion to host cells and tissues, disruption of mucosal innate and adaptive immunity, and evasion of mucociliary clearance. Targeting mucosal innate immunity and epithelial microinvasion while inducing adaptive immune responses may effectively prevent pneumococcal colonization and disease.^367^

Salmonella is another classic example of a pathogen exploiting host immune responses. The disease caused by this pathogen depends on its serotype and the individual characteristics of the host. Non-typhoidal Salmonella gastrointestinal infections are caused by hundreds of different serotypes within the species.^368^ Despite a strong innate immune response from the host, non-typhoidal Salmonella serotypes can extensively colonize the intestine and are continuously shed in the feces of the infected individual for up to a month. Consistent with these clinical observations, several studies have shown that Salmonella can exploit the competition between gut inflammation and resident microbiota to proliferate and grow in the inflamed intestinal environment.^369–373^ Additionally, naturally acquired antibodies against non-typhoidal Salmonella have been reported to reduce the risk of disease caused by this pathogen.^374^

Respiratory syncytial virus (RSV) is a ubiquitous respiratory pathogen, a major cause of lower respiratory tract infections, bronchiolitis, and pneumonia in infants, the elderly, and immunocompromised individuals, with a prevalence comparable to that of influenza. RSV primarily invades airway epithelial cells and replicates intracellularly, leading to necrosis, shedding of epithelial cells, and mucus sloughing, accompanied by the release of numerous inflammatory mediators. This process triggers immune and pathological responses, disrupting the structure and function of the airway epithelium, which is a key step in the development of related diseases. Despite over 60 years of research, RSV remains a major infectious disease lacking an effective vaccine. Currently, two RSV vaccines’ Biologics License Applications (BLA) have been accepted by the FDA, with one receiving fast-track designation. Additionally, although monoclonal antibodies developed by AstraZeneca and Sanofi are not preventive vaccines, they can provide RSV immune protection without activating the immune system.

Influenza viruses can cause respiratory infections ranging from mild to severe. Seasonal influenza viruses, including subtypes H1N1 and H3N2 of influenza A and influenza B viruses, result in approximately 3 to 5 million cases of severe illness and 290,000 to 650,000 deaths worldwide each year1,2. Additionally, avian influenza viruses such as H5N1 and H7N9 can cause zoonotic infections. Influenza viruses enter the host through mucosal surfaces, where the hemagglutinin (HA) on the virus particles binds to sialic acid residues on the mucosa.^375^ This binding mechanism is thought to be part of the natural defense of host cells.^376^ The role and mechanisms of mucosal antibodies against influenza virus antigens require further research to develop vaccines capable of inducing potent mucosal immune responses. Although the drivers of long-term immunity remain unclear, antibody responses from natural influenza virus infections are generally broader and more durable than those induced by influenza vaccines. Understanding these mechanisms is crucial for designing vaccines that provide long-term protection.

Herpes simplex virus (HSV) is a member of the herpesvirus family, divided into HSV-1 and HSV-2, causing oral and genital herpes, respectively. The herpesvirus entry mediator (HVEM), a member of the tumor necrosis factor receptor (TNFR) family, is a critical receptor in the HSV infection process, aiding in viral entry into host cells. HVEM not only plays a significant role in viral invasion but also in mucosal immunity. Recent studies indicate that HVEM’s function is particularly crucial in colitis, with its role in epithelial cells being vital for innate mucosal defense against pathogenic bacteria in mouse models of colitis.^377^ HVEM enhances immune responses and increases the expression of immune-related genes in epithelial cells by activating the NF-κB signaling pathway and promoting kinase-dependent activation of Stat3, indicating its important coordinative role in mucosal immunity. Therefore, targeting HVEM agonists may help improve mucosal immune defenses and enhance resistance to infections.

Human immunodeficiency virus (HIV) is a lentivirus primarily transmitted through mucosal surfaces during sexual contact but can also be spread through blood and vertical transmission. The vast majority of global infections are caused by HIV-1, while HIV-2 is mainly confined to West Africa and is generally less pathogenic.^378^ Primary infection may present as seroconversion illness with nonspecific systemic symptoms, most of which are self-limiting. However, chronic infection with HIV-1 is characterized by persistent viral replication and gradual depletion of CD4 + T cells. After approximately ten years, infected individuals progress to acquired immunodeficiency syndrome (AIDS), becoming susceptible to opportunistic infections and malignancies. The mucosal immune system is a primary target of HIV infection, thus playing a crucial role in AIDS progression. Early significant depletion of gut-associated lymphoid tissue (GALT) leads to mucosal disruption and increased microbial translocation, resulting in chronic immune activation and dysfunction, further driving AIDS progression. Additionally, AIDS-defining illnesses often occur at mucosal sites, directly resulting from compromised mucosal immunity. For example, Pneumocystis jirovecii pneumonia, an opportunistic fungal infection frequently causing death in AIDS patients, is characterized by impaired innate and adaptive immune responses in the lung mucosa. Other important mucosal opportunistic infections include esophageal or respiratory candidiasis, HCMV-related gastrointestinal diseases, chronic mucosal ulcers due to HSV reactivation, and chronic intestinal isosporiasis.

### Mucosal immunity against SARS-CoV-2 infection

**Fig. 5:**
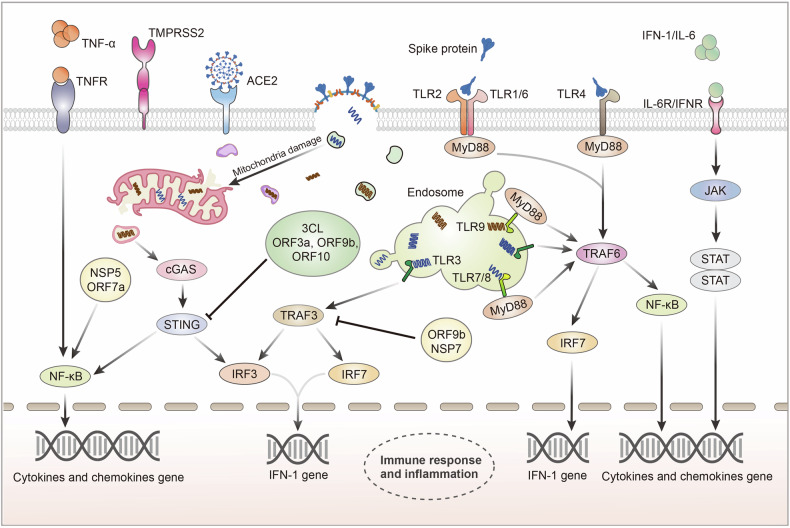
Immune signaling pathway of infected cells. Transmembrane protease serine 2 primes the severe acute respiratory syndrome coronavirus-2 (SARS-CoV-2) spike (S) protein. The activated S protein binds to angiotensin converting enzyme-2 (ACE2), resulting in membrane fusion. Thereafter, SARS-CoV-2 RNA chain invades the cells and replicates. The binding of ACE2 to the S protein is blocked, resulting in mitochondrial damage and mitochondrial DNA (mtDNA) leakage. S proteins can activate Toll-like receptor (TLR) 2 and 4 on the cell surface, whereas ssRNA and CpGDNA can activate endosomal TLR7/8 and TLR9, thereby activating the nuclear factor kappa light chain enhancer of activated B cells (NF-κB) pathway and TRAF6 by MyD88 dependent pathway. TLR3 binds to dsRNA and activate TRAF3 and TRAF6 by MyD88 independent pathway, thereby activating interferon regulating factor (IRF) 3 and 7. MtDNA activates the cGAS-STING pathway, which in turn activates the NF-κB pathway and IRF3. NF- κB pathway regulates the expression of various cytokines and chemokines, whereas IRF3 and IRF7 regulate the expression of type I interferon. Secreted tissue-necrosis factor-alpha (TNF- α) binding to TNF receptor cell surface activates the TNF pathway and enhances the NF- κB pathway. Interferon-1 and Interleukin-6 bind to responsive receptors to activate the JAK/STAT pathway, thereby promoting the secretion of proinflammatory factors. SARS-CoV-2 accessory proteins participate in the regulation of cellular immune signal transduction. Non-structural protein (NSP)-5 and ORF7a can activate NF- κB pathway; 3CL, ORF3a, ORF9b, and ORF10 can inhibit the activation of SRING protein, whereas ORF9b and NSP7 can inhibit the activation of TRAF3

Once epithelial cells are infected, intracellular and intercellular immune signaling pathways are activated, leading to local inflammation. If immune responses are rational and controllable, coronavirus is eliminated efficiently and rapidly, whereas dysregulated immune responses may elicit hyperinflammation, enhancing immune-mediated pathology.^379^ Therefore, it is important to thoroughly comprehend how immune signaling pathways respond to virus. Therefore, we attempted to construct an intracellular innate immune signaling pathway network based on the recognized patterns of origin and downstream pathways’ cascade reactions to link the function of immune cells with inflammation (Fig. 5).

One of PRRs, TLRs, widely exist epithelial and tissue-resident immune cells that recognize pathogen-associated molecular patterns (PAMPs) and initiate innate immunity.^380^ As conserved upstream signaling proteins, TLRs can identify components of SARS-CoV-2 as PAMPs. Cell surface TLRs are likely to bind to SARS-CoV-2 structural proteins. Khan et al. found TLR2 sense S protein in lung epithelial cells, and then dimerize with TLR1 or TLR6 to activate the MyD88 dependent NF-κB pathway.^381^ Other than S protein, envelope (E) and N proteins can also be sensed by TLR2; however, only the N protein can induce NF-κB pathway like the S protein,^382^ whereas in experiments on E protein, inflammatory substances are merely present.^383^ TLR4 mediates immune responses against gram-negative bacteria by recognizing bacterial lipopolysaccharides.^384^ In THP-1 cells, a cell line of monocytes, TLR4 exhibits a comparable affinity to the spike trimer.^385^ Moreover, the S1 subunit also activates TLR4 signaling of macrophages that subsequently elicits proinflammatory responses.^386^ Intracellular TLRs bind to nucleic acid fragments or accessory protein of SARS-CoV-2. After releasing its contents into the infected cell, the ssRNA of the coronavirus can be detected by endosomalTLR7/8,^387,388^ and during replication, dsRNA appears and is recognized by TLR3.^389^ SARS-CoV-2 infection of Calu-3/MRC-5 multicellular spheroids model can both activate two RNA sensor pathways, and thus on the one hand, TLR7/8 signaling triggers the IRF-7 and MyD88-NF-kB signaling pathways which bring about the release of proinflammatory cytokines and type I IFNs;^389,390^ on the other hand, TLR3 signaling relies on the interaction between TRIF, TRAF6, and TRAF3, leading to the activation of IRF3, increasing the production of IFN-α and IFN-β.^391^ Apart from RNA, an accessory protein encoded by ORF9b and non-structural protein 7 (NSP7) also suppress the induction of type I and III IFNs by TLR3-TRIF signaling.^392,393^ Additionally, as a DNA sensor, TLR9 recognizes the CpG-rich DNA fragments,^394^ and seems unlikely to be activated by the positive-sense ssRNA virus; however, SARS-CoV-2 infection can indirectly initiate TLR9 signaling. In human umbilical vein endothelial cells, SARS-CoV-2 infection promotes mitochondrial dysfunction and increases mitochondrial membrane potential, which lead to the leak of mitochondrial DNA (mtDNA) to cytoplasm, and then mtDNA binds to TLR9, triggering inflammatory responses that lead to vascular dysfunction.^395^ Thus TLR9 activation contributes to the deterioration of COVID-19, and TLR9 is considered an inhibitory target for COVID-19 treatment.^395,396^ But if TLR9 is stimulated in advance or as an adjuvant in vaccine design, it has distinctly opposite effect to protect against coronavirus infection.^397^

Damage-associated molecular patterns (DAMPs) are endogenous molecules that participate in TLR signaling.^398^ Cells damaged or killed by coronavirus discharge DAMPs (mainly nuclear or cytosolic proteins) into the extracellular space and TLRs on surrounding cells identify them, leading to the loss of control and development of cytokine storm syndrome (CSS).^398,399^ TLRs are receptors capable of recognizing coronavirus infection, TLRs are involved in the first step of activating immune signaling pathways. Immune signaling pathways regulate immune responses against infections. NF-κB hikes up the induction of various proinflammatory cytokines (such as IL-1, IL-2, IL-6, IL-12, TNF-α, IFN-α, IFN-β, and GM-CSF) and chemokines (such as IL-8, MIP-1, MCP1, RANTES, and eotaxin), and engage in inflammasome regulation.^400^ All of SARS-CoV-2 induced TLR signaling can initiate the NF-κB pathway,^401^ but except for TLR signaling, NSP5 can upregulate SUMOylation of the mitochondrial antiviral-signaling protein,^402^ and ORF3a can enhance IKKβ-NEMO (IκB kinase-NF-κB essential modulator) interaction,^403^ both of which in an alternative way activate NF-κB pathway. Due to the cross-linking effect of NF-κB pathway, TNF-α pathway and JAK/STAT pathway are promoted consequently, forming a positive feedback loop. Above all, TNF-α combines with TNFR and strengthens the NF-κB pathway,^404^ whereas IL-6 enhances the JAK/STAT pathway, increasing the proinflammatory factors independently.^405,406^ Moreover, the cGAS-STING pathway activated by released mtDNA facilitates the expression of type I IFN genes in COVID-19.^407^ Conversely, 3CL, ORF3a, ORF9b, and ORF10 of SARS-CoV-2 directly connect with STING and suppress the IFN response.^392,408,409^

In conclusion, the immune signaling pathway network is activated and performs anti-coronavirus activity; however, CSS occurs because of the dysregulation and positive feedback of proinflammatory factors.^410^ As a result of the immune system overreactions, CSS macroscopically brings about acute respiratory distress syndrome and even respiratory failure,^68,410^ and microscopically, infiltration of lymphocytes results in tissue damage and magnifies infection.^411^ Meanwhile, coronavirus may inhibit the immune signaling pathway to weaken the anti-virus competence of host cells. Therefore, understanding immune signaling pathways aids in proposing pertinent strategies for COVID-19.

### Mucosal immunity in gastrointestinal disease

Crohn’s disease (CD), an inflammatory bowel disease (IBD), is characterized by polymorphisms in risk genes, discontinuous penetrating inflammation of the gastrointestinal mucosa, and disturbances in the gut microbiota.^412–414^ Jain et al. identified a fungus, *Debaryomyces hansenii*, which impairs mucosal wound healing and dominates the fungal community in intestinal wounds in mouse models and inflamed mucosa of CD patients.^415^ Recent findings have further revealed that common gut commensal and food-derived yeasts serve as direct activators of altered CD4^+^ T cell responses in CD patients.^416^ This underscores the role of yeasts as significant contributors to dysregulated immune responses and sustained mucosal inflammation in CD. Together with Jain et al.‘s findings, this study underscores the critical need for comprehensive investigation of mucosal biopsy samples from patients across varying severities and progression stages of CD or other forms of IBD, aiming to elucidate how specific microorganisms contribute to mucosal pathology in these patients. Although yeasts play a role in IBD pathogenesis, it is widely accepted that IBDs are primarily driven by or result from alterations in the intestinal bacterial microbiota. For example, recent studies have illuminated the role of bacterial dysbiosis in IBD, wherein specific bacterial taxa are either depleted or overrepresented, culminating in immune dysregulation and chronic inflammation. Notably, studies have demonstrated that a reduction in *Faecalibacterium prausnitzii*, a bacterium known for its anti-inflammatory properties, correlates with disease severity in CD patients.^417^ Furthermore, additional research has identified a bloom of adherent-invasive *Escherichia coli* (AIEC) in the ileum of CD patients, which is linked to exacerbated inflammation and altered immune responses.^358^ These findings emphasize the intricate interplay between fungal and bacterial constituents within the gut microbiota and their collective impact on IBD pathogenesis.

Chronic inflammation and tissue damage are key features of inflammatory diseases like Crohn’s disease and ulcerative colitis, highlighting the critical role of the mucosal immune system in maintaining intestinal mucosal integrity and immune homeostasis. Dysregulation of immune cell activity and inflammatory mediator release can contribute to the development and progression of inflammatory diseases. The homeostasis that the gastrointestinal mucosal immune system maintains far beyond the gastrointestinal tract itself, gastrointestinal mucosal immunity is also associated with autoimmune diseases such as rheumatoid arthritis (RA), systemic lupus erythematosus (SLE) and type-1 diabetes (T1D).^418–420^ Commonly, their pathogeneses are still unclear, but they are all related to the loss of intestinal mucosal barrier integrity mediated by intestinal inflammation and gut microbiota, which implies the importance of the intestinal mucosal barrier in overall health. Current research emphasizes the significant impact of mucosal immunity on regulating host immunological balance through interactions with bioactive metabolites originating from the gut microbiome, specifically conjugated linoleic acids (CLAs). These CLAs play a role in modulating a specific subset of CD4^+^ intraepithelial lymphocytes in the small intestine by influencing the transcription factor HNF4γ.^421^ IgA plays an important role in mucosal biology, and the relatively mild symptoms of most patients with selective IgA deficiency (SIgAD) have been a clinical challenge. A study has shown that mucosal and systemic antibodies work together to target specific gut microbes to achieve immune homeostasis. In patients with SIgAD, the lack of mucosal IgA allows certain gut bacteria to trigger an abnormal immune response, leading to symptoms and immune dysregulation.^422^ IgA nephropathy is the predominant glomerular disease observed in kidney biopsies of individuals with inflammatory bowel disease (IBD), with emerging research indicating that mucosal immunity plays a significant role in its pathogenesis.^423^ The potential contribution of MALT, specifically gut-associated lymphoid tissue and nasopharyngeal-associated lymphoid tissue, to the development of IgA nephropathy warrants further investigation.

### Mucosal immunity in autoimmune diseases

The relationship between mucosal immunity and autoimmune diseases is significant. Autoimmune diseases, characterized by the immune system attacking the body’s own tissues, encompass conditions such as rheumatoid arthritis, IBD, and systemic lupus erythematosus. Dysregulation of the mucosal immune system can contribute to the pathogenesis of autoimmune diseases by exacerbating immune responses against normal tissues. RA is a disabling autoimmune and inflammatory disease that means the immune system attacks the joints by mistake. One hypothesis for the pathogenesis is that aberrant intestinal mucosal function increases intestinal permeability, inducing the transfer of immune cells to the synovial joints.^424^ In the murine collagen-induced arthritis (CIA) model, the standard RA model used in preclinical research, obvious gut microbiota dysbiosis and intestinal mucosal inflammation occur in the early stage.^425^ Besides, themes targeting intestinal mucosa have been proven to work. Tajik N. et al. reported that butyrate and a cannabinoid type 1 receptor agonist could alleviate intestinal barrier permeability thus inhibiting arthritis development.^426^ Furthermore, directly aimed at zonulin, a potent regulator for intestinal tight junctions, the zonulin antagonist larazotide acetate effectively reduces arthritis onset.^426^ What’s more, targeting metabolic disorders caused by microbiota dysbiosis, 3,3-dimethyl-1-butanol is protective in the CIA model.^427^ Similarly, an imbalance in the gut microbiota was observed in SLE patients; *Enterococcus gallisepticum* could be detected in patient-derived hepatic tissue, which suggested that pathogenic agents (lipopolysaccharide or bacteria) may cross the intestinal mucosal barrier, accelerating SLE.^428,429^ In gut leakage-induced lupus murine models, the observed production of anti-double-stranded DNA antibodies and the deposition of circulating immune complexes intensified the disease,^430^ indicating the increased permeability of the intestinal tract enhances the progression of lupus. T1D is an autoimmune disease as a consequence of auto-reactive T cells attacking β-cells in the pancreas. The disruption of the intestinal mucosal barrier in non-obese diabetic mice stimulates the activation and migration of islet-reactive T cells,^431^ and it was thought as a trigger for T1D. The regulator zonulin plays a significant role in T1D. The high serum concentration of zonulin was found in diabetic mouse and rat models,^432,433^ so zonulin antagonists may be considered as a treatment option. T1D patients often present intestinal inflammation, and the decline of IL-17A, IL-22 and IL-23A are conserved in T1D mice.^434^ Microbiome alternation or translocation is also a critical push in the development of T1D.^435,436^

### Mucosal immunity in cancers

Moreover, research has demonstrated a correlation between mucosal immunity and specific types of cancer. Chronic inflammation and immune dysregulation have been identified as potential factors that could elevate the susceptibility to certain cancers, particularly those affecting mucosal tissues like colon and stomach cancers. Consequently, preserving the integrity of the mucosal immune system may play a crucial role in the prevention of specific cancer types. Lynch syndrome (LS) is the most common hereditary colorectal cancer (CRC) syndrome with a significant immune infiltrate due to high levels of immunogenic shift code neoantigens. However, the normal colonic mucosal immune status of patients with LS is not well characterized. Recent studies have shown that a normal mucosal immune profile may be an important modifier influencing the development of colorectal tumors in LS patients, highlighting the importance of mucosal immune surveillance for colorectal tumor risk assessment in LS patients.^437^ mRNA cancer vaccines represent a novel immunotherapeutic strategy; however, their efficacy in colorectal cancer is limited due to insufficient activation of the mucosal immune response. A newly developed mRNA cancer vaccine co-delivering all-trans-retinoic acid (ATRA) and mRNA via lipid nanoparticles (LNP) significantly enhances mucosal immune responses and cytotoxic T cell infiltration, resulting in improved tumor inhibition and prolonged survival in colorectal cancer models.^438^

## Prevention and treatment therapies based on mucosal immunity

**Fig. 6:**
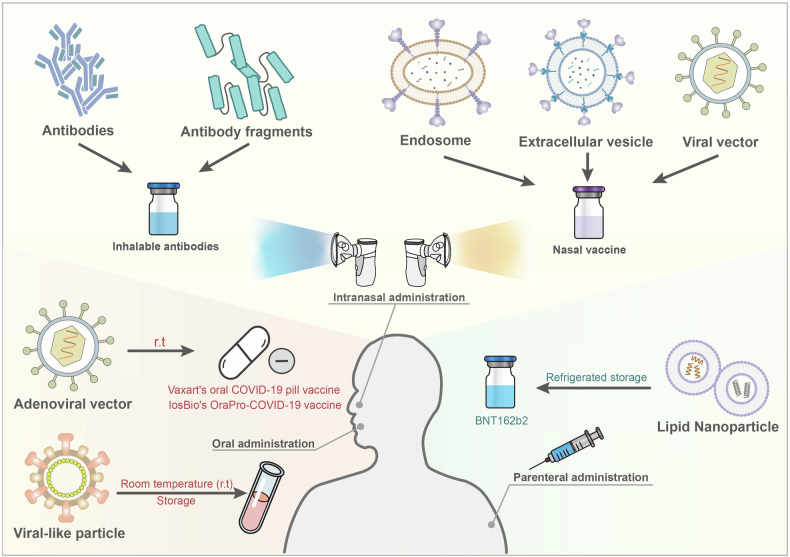
Novel therapies and their administration. Drug/vaccine route of administration: nasal, oral, and parenteral administration. Most conventional coronavirus disease 19 vaccines require cold storage and intramuscular administration to activate systemic immunity, such as BNT162b2, an RNA vaccine packaged with lipid nanoparticles. Mucosal vaccines are administered intranasally or orally. Oral vaccines targeting sublingual and intestinal mucosal immunity are usually stored at room temperature because they can withstand harsh conditions in the gastrointestinal tract. Vaxart and IosBio oral vaccines, both adenovirus vectors, have been developed into capsules and pills for ease of administration. Recombinant oral vaccines based on virus-like particles, can also be stored at room temperature in liquid form. Function of intranasal vaccines in the respiratory tract: viruses, exosomes, and extracellular vesicles can be used as vectors to carry the severe acute respiratory syndrome coronavirus-2 (SARS-CoV-2) spike protein or its optimized gene codes. Intranasal vaccines are atomized into small droplets using a nebulizer and inhaled, exerting their effects on the nasal cavity and airway. Inhaled antibodies are used as therapeutic agents. They have instant effects because inhaled neutralizing antibodies or antibody fragments are directed to the respiratory tract, where SARS-CoV-2 is rampant, neutralizing viruses and inhibiting their replication

The primary processes of initiating systemic immunity in humans or targeting the vital movement of coronavirus are widely applied in developing vaccines and miracle drugs for COVID-19. BNT162b2 vaccine and Paxlovid have been widely employed and have shown striking results against COVID-19. The former applies mRNA fragments that carry virus antigen information to cells for duplication, thereby stimulating both cellular and humoral immunity against an infection^439,440^ (Fig. 6). The latter is an inhibitor of an essential protease in SARS-CoV-2 replication, M^PRO^ to intercept its amplification.^441,442^ The strategic significance of mucosal immunity has been realized as it is a frontline defense against coronavirus. An increasing number of therapies based on mucosal immunity have been proposed and proven effective. Two of such are mucosal vaccines and inhalable antibodies. While the diseases or viruses we encounter may vary, the human mucosal immune system remains stable. These targeted strategies can equally be applied to other diseases or unknown viruses.

### Mucosal vaccines

In contrast to conventional or novel injectable vaccines that mainly activate systemic immune responses, mucosal vaccines specifically trigger robust adaptive immune responses at the effector sites to forestall infection for the first time, instead of restricting infection and retarding the development of diseases, owing to its distinct delivery systems.^443,444^ After mucosal vaccination, induced SIgA could migrate to distant induction sites, forming a defense network against pathogens.^445^

Early attempts at mucosal vaccines are on oral vaccination, among which the most significant milestone was the development of the oral polio vaccine by Dr. Albert Sabin in the 1960s.^446^ Poliovirus (PV), first discovered in 1909, is the virus that causes poliomyelitis, an acute infectious disease. The virus often invades the central nervous system and damages motor neurons in the anterior horn of the spinal cord. The virus spreads along the afferent nerve pathway in the brain because of its specific affinity for the neuronal cell receptor CD155. Eventually, the virus damages the motor nerve cells of the anterior horn of the spinal cord, leading to flaccid paralysis of the limbs. The American doctor Jonas Salk succeeded in developing an inactivated polio vaccine. Around the same time, American microbiologist Albert Sabin developed an oral live attenuated polio vaccine. Today, both the inactivated poliovirus vaccine (IPV) and the live attenuated oral poliovirus vaccine (OPV) are widely used worldwide and play an important role in the effective control of poliovirus epidemics. The live attenuated oral polio vaccine (OPV) is a mucosal vaccine that activates the immune response of the gastrointestinal mucosa through oral administration without injection and has a higher antiviral protective efficacy than the injectable inactivated vaccine. In addition to the oral polio vaccine, eight oral vaccines are currently 2licensed for the prevention of cholera, salmonella and rotavirus. Live attenuated influenza vaccine remains the only licensed intranasal vaccine.

#### Intranasal administration

Intranasal administration is a common delivery approach to the respiratory mucosa (Fig. 6). Mucosal vaccines spread throughout the respiratory tract when instilled or inhaled; thus, tissue-resident lymphocytes are stimulated and immune responses are elicited. Most reported mucosal vaccines are virus-vectored and recombinant, or sometimes unadjuvanted S proteins are directly administered intranasally.^447^ Engineered adenoviral, lentiviral and pediatric parainfluenza viral vectors are used to encode the S protein or its subunit,^448–453^ and other viral proteins are considered to enhance the immunogenicity of vaccines.^454^ What’s more, biodegradable amine-co-ester polyplexes are used to deliver mRNA, and intranasal vaccination achieves high transfection of mRNA in the lung.^455^ After vaccination, bodies can produce attenuated SARS-CoV-2 proteins in the respiratory tract, which are recognized as antigens by the mucosal immune system. Furthermore, in recombinant mucosal vaccines, the S protein or its RBD is directly transported to the respiratory tract. Therefore, the delivery systems largely determine the effectiveness of recombinant mucosal vaccines. The RBDβ-HR self-assembled trimer vaccine has been developed to target emerging SARS-CoV-2 variants, including Omicron.^456^ This vaccine incorporates the RBD of the Beta variant and heptad repeat subunits, demonstrating robust inhibitory activity against RBD-hACE2 binding across various viral variants. It elicits high titers of specific binding antibodies and cross-protective neutralizing antibodies against Omicron and other major variants, while promoting a broad cellular immune response involving T follicular helper cells and activated T cells.^456^

Furthermore, exosomes are suitable containers for packaging vaccines like recombinant protein and mRNA dry powder because they are easy to deliver and endogenic.^457,458^ Moreover, bacteria-derived extracellular vesicles decorated with an RBD apply to vaccine delivery and are proven valid against SARS-CoV-2.^459^ Mucosal vaccines can improve mucosal immunity, reduce inflammatory infiltration, and elicit systemic immunity, thereby offering durable protection against coronavirus and its variants. Recent research shows that intramuscular mRNA and inactivated vaccines fail to boost mucosal IgA responses adequately, especially against Omicron variants. However, intranasally administered dimeric or secretory IgA antibodies, like DXP-604, demonstrate significantly better neutralizing activity and offer strong protection, presenting a promising approach to combat SARS-CoV-2 variants.^460^ In addition to coronaviruses, influenza virus vaccines can also induce mucosal immunity, and a replication-competent adenovirus-vectored influenza vaccine induces durable systemic and mucosal immunity.^461,462^

In addition to nasal administration, tracheal administration is also excellent for inducing mucosal antibody and T-cell responses. Intratracheal administration of a bivalent Ad26-based SARS-CoV-2 vaccine induces strong mucosal and cellular immunity, providing almost complete protection against SARS-CoV-2 BQ.1.1 challenge.^463^ Intranasal vaccines serve a crucial role in the respiratory tract by using viruses, exosomes, and extracellular vesicles as vectors to deliver the SARS-CoV-2 spike protein or its optimized genetic codes. These vaccines are atomized into fine droplets using a nebulizer and inhaled, targeting the nasal cavity and airway. Inhaled antibodies function as therapeutic agents by providing immediate effects. When neutralizing antibodies or antibody fragments are inhaled, they are directed to the respiratory tract, a primary site of SARS-CoV-2 infection, where they neutralize the virus and inhibit its replication (Fig. 6).

#### Oral administration

Regarding gastrointestinal mucosal immunity, oral administration is a common method that targets cells on the mucosal surfaces under the tongue or in the gastrointestinal tract (Fig. 6). However, unlike in the respiratory tract, the higher temperature, acidic environment in the stomach, and longer and rugged delivery routes in the gastrointestinal tract require further consideration during oral administration. Bellier B. et al. developed a thermostable oral vaccine via virus-like particle delivery in mouse and hamster models.^464^ In addition to the S and M proteins, this vaccine employed various surface proteins from an intestinal protozoa, Giardia lamblia, contributing to its degradation resistance. Two other oral vaccines, Vaxart’s oral COVID-19 pill vaccine and IosBio’s OraPro-COVID-19 vaccine (Fig. 6), are in the clinical trial phase; both vaccines are adenoviral-vectored targeted at S and N proteins.^465^ The N protein is less prone to mutations (more conserved) and is strongly immunogenic to T cells. Therefore, these vaccines can effectively tackle the immune escape of various variants and trigger a superior T-cell response. All these oral vaccines are engineered to withstand hostile conditions and body temperature without losing efficacy, and the pill or capsule form makes them easier to store and transport. Recently, a strategy to effectively concentrate immunogens and adjuvants in gut-draining lymph nodes (LNs) could induce gut-associated mucosal immunity. A lymph-targeting nanoemulsion vaccine formulation effectively concentrates immunogens and adjuvants in gut-draining lymph nodes, inducing robust gut-associated mucosal immunity with significantly higher antigen-specific IgG and IgA titers and strong neutralizing antibody responses against SARS-CoV-2.^466^

Considering the limitations of using injectable vaccines for inducing mucosal immunity compared to systemic immunity,^467–469^ mucosal vaccines can be used as a supplement in the vaccination program to reactivate mucosal immunity, prevent virus transmission, and offer mass protection against variants of concern.^450,470^ Therapies that combine mucosal and injectable vaccines have been proposed in several studies and reviews.^447,471^ Although a booster dose of the injectable COVID-19 vaccine activated mucosal immunity as mucosal vaccines,^467,472–474^ mucosal vaccines have irreplaceable properties. First, intranasal or oral administration is noninvasive and simple, which confers high patient compliance and low production and distribution costs for mucosal vaccines. Second, it is not in high demand as injectable vaccines by healthcare workers, and there is no professional requirement for oral pill or capsule vaccines. Moreover, unlike the acute responses and latent side effects associated with systemic immunity, mucosal vaccines targeted at mucosal immunity are less reactive and safer.^475,476^ Finally, mucosal vaccines function directly and swiftly at mucosal effector sites, whereas other vaccines such as BNT162b2 often lead to the delayed activation of mucosal immunity.^443,473^

#### Approved or in-trial mucosal vaccines

Nowadays, more and more administration routes have been put forward, and numerous mucosal vaccines have been invited to prevent various diseases. Here, we list approved or in-trial mucosal vaccines (Table 2),^477–484^ and these vaccines lean toward traditional concepts of vaccine, for most of them are attenuated viruses. FluMist Quadrivalent and FluGen’s M2SR are nasal vaccines containing live attenuated influenza virus strains, with the former inducing cross-reactive SIgA and the latter designed for single-round replication by deleting part of the M2 gene.^477,478^ The Ad4-H5-Vtn vaccine uses an adenovirus vector encoding recombinant haemagglutinin and targets both the nasal cavity and rectum.^479^ SynGEM is another nasal vaccine, incorporating RSV F protein with an immunostimulatory particle.^480^ The oral Sabin mOPV and Vivotif vaccines consist of live attenuated poliovirus and Salmonella typhi Ty21a strain, respectively, targeting the gut.^481^ Rotarix and RotaTeq, oral vaccines against Group A rotaviruses, utilize live attenuated strains, with RotaTeq also featuring recombinant surface proteins.^483^ Dukoral and Vaxchora, oral vaccines against Vibrio cholerae, contain inactivated bacterial strains and recombinant cholera toxin B subunit.^484^ Additionally, the CysVac2/Advax nasal vaccine employs a multistage fusion protein targeting Mycobacterium tuberculosis in the lungs. These vaccines leverage advanced delivery systems and genetic modifications to enhance immune responses, demonstrating significant progress in the field of vaccinology.

**Table 2:** Approved or in-trial mucosal vaccines for other diseases

Name	Type	Characters	Targeted pathogens	ref.
**FluMist Quadrivalent**	Nasal vaccine	Consists of each two strains of live attenuated influenza virus A and B, induces cross-reactive SIgA, nasal cavity as the induction site;	Influenza virus	^490^
**FluGen’s M2SR vaccine**	Nasal vaccine	Consists of both live attenuated influenza virus A and B types, deletes a portion of the M2 gene resulting in a single round of viral replication,	Influenza virus	^491^
**Ad4-H5-Vtn**	Oral/nasal vaccine	Adenovirus-vectored, encodes recombinant haemagglutinin of influenza virus, nasal cavity and rectum as the induction site;	Influenza virus	^492^
**SynGEM**	Nasal vaccine	Consists of RSV F protein with an immunostimulatory bacterium-like particle, nasal cavity as the induction site;	Respiratory syncytial virus	^493^
**Sabin mOPV**	Oral vaccine	Consists of a monovalent live attenuated poliovirus, gut as the induction site;	Poliovirus	^494^
**CysVac2/Advax**	Nasal vaccine	Consists of multistage fusion protein CysVac2, Lung as the induction site;	Mycobacterium tuberculosis	^495^
**Vivotif**	Oral vaccine	Consists of a live attenuated Salmonella typhi Ty21a strain, gut as the induction site;	Salmonella typhy	^496^
**Rotarix**	Oral vaccine	Consists of a live attenuated human rotavirus RIX4414 strain, gut as the induction site;	Group A rotaviruses	^497^
**RotaTeq**	Oral vaccine	Live rotavirus strains containing recombinant surface proteins G1, G2, G3, G4 and P1(8) of human-bovine reassortant rotaviruses;	Group A rotaviruses	^498^
**Dukoral**	Oral vaccine	Consists of inactivated strains of Vibrio cholerae serotype O1 and recombinant cholera toxin B subunit, gut as the induction sites;	Vibrio cholerae	^499^
**Vaxchora**	Oral vaccine	Consists of a weakened human rotavirus RIX4414 strain, gut as the induction sites;	Vibrio cholerae	^500^

#### Challenges and strategies in the development of mucosal vaccines

The development of novel preventive and therapeutic strategies targeting mucous membranes faces significant challenges. Mucosal vaccines are particularly affected by the biological barriers of mucosal tissues, which include not only physical barriers but also an immunosuppressive microenvironment, variable pH levels, and a diverse microbiota capable of rapidly clearing and degrading exogenous substances. Consequently, mucosal vaccines, irrespective of their immunogenic composition, need to prevent degradation by superficial mucosal tissues, resist mechanical clearance, and penetrate the mucus layer until they reach the target location and are taken up by the appropriate cell populations. This process is followed by the activation of immunity through presentation by immunogenic APCs. Addressing these challenges is crucial for the successful development of effective mucosal vaccines. Researchers have proposed various innovative vaccine strategies, such as using nanoparticles and hydrogels to enhance vaccine efficacy (Fig. 6). Cationic liposomes, studied for their adjuvant properties, have emerged as effective carriers for gastrointestinal vaccines. Cationic liposomes encapsulating DNA vaccines encoding Mycobacterium tuberculosis proteins induce antigen expression in the intestinal epithelium, M cells, dendritic cells, and Peyer’s patches of mice.^485^ Viral vectors, engineered to deliver genetic material (DNA or mRNA) encoding target antigens in a manner that mimics natural infection, are among the most effective platforms for breaching mucosal barriers and eliciting robust immune responses at mucosal entry sites. Vaxart has developed a thermostable oral enteric-coated tablet containing a replication-deficient recombinant adenovirus type 5 (Ad5) vector vaccine. This vaccine incorporates two payload genes encoding pathogen-specific protein antigens and a double-stranded RNA (dsRNA) adjuvant on the same viral vector. Clinical trials have shown that this carrier induces potent mucosal and systemic immunity against a range of enteric and respiratory pathogens.^454^ Virus-like particles (VLPs) represent another promising candidate for effective oral antigen delivery. Oravax, a VLP-based trivalent oral vaccine, targets three SARS-CoV-2 surface proteins and is encapsulated in a protective capsule. Preclinical studies have demonstrated its safety and efficacy in eliciting IgG and IgA responses, and it is poised to enter Phase 1/2 clinical trials.^486^

Actually, novel nanoparticle vectors have been successfully applied to mucosal vaccines against other diseases, and the conceptions may inspire coronavirus mucosal vaccine design. Lipid nanoparticles (LNPs) are widely used in vaccine development because of excellent diffusion, stability, and accessibility in vivo (Fig. 6). Valentina B. et al. fused the ectodomain of the influenza M2 protein with cholera toxin-derived adjuvant protein and chose LNPs to load them.^487^ This vaccine elicits superior mucosal immune responses against influenza virus infection in mouse models. Likewise, Hartwell B. L. proposed amph-proteins that immunogens are modified with an amphiphilic albumin-binding polymer-lipid tail to conjugate lipids,^488^ which enhance antigen transmucosal uptake in HIV and SARS-CoV-2 cases. However, the highly inflammatory nature of LNPs merits further assessment before clinical trial.^489^ Other nano biomaterials like chitosan and copolymer poly methyl vinyl ether/maleic anhydride are also utilized in mucosal vaccines against influenza virus and *Shigella flexneri* respectively.^490,491^ Aside from the above nanomaterials, pH-responsive nanoparticle vaccines can achieve targeted regulation. For example, the endosomolytic copolymer comprising of propylacrylic acid, butyl methacrylate, and dimethylaminoethyl methacrylate can protect inner antigens as shells, but after cellular uptake of APCs, it senses endosomal acidification and exposes core antigen of vaccinia virus or influenza virus and nucleic acid adjuvant.^492^ Also, dependent on the pH discrepancy of the mucosal surface, poly DL-lactic-co-glycolic acid nanoparticles encapsulating a peptide or protein manage to directionally induct T cell immunity against HIV on rectal and vaginal mucosa.^493^ Furthermore, metal nanoparticles like nano-silver and nano-iron are regarded as ideal adjuvants in mucosal vaccines.^494,495^ An intranasal mosaic RBD nanoparticle vaccine shows promise as a pan-sarbecovirus vaccine by inducing strong mucosal immunity and broad protection against SARS-CoV-2 variants.^496^ Parenteral vaccines often fail to induce strong cellular and mucosal immunity. A proposed subcutis-to-intestine cascade (LUCID) using nanovaccines effectively enhances these immune responses.^497^ Additionally, a microneedle patch delivering chitosan-encapsulated DNA vaccines induces robust systemic and mucosal T cell immunity, comparable neutralizing antibodies, and can be stored at room temperature, offering an easy-to-administer and thermostable alternative to traditional vaccines.^498^ Last but not least, other than secretory antibodies and tissue-resident T cells, there are still potentially efficacious components of the mucosal immune system, such as DCs, macrophages, and innate lymphoid cells, which can be further investigated and applied to vaccine development, and more delivery systems and adjuvants can be considered to optimize the performance of vaccines.^2,443^ Furthermore, analyses of the immunological memory of mucosal vaccines, which are instructive for formulating doses and schedules of vaccines, are indispensable but lacking.^499^

### Inhalable antibodies

Monoclonal antibody (mAb) therapy represents a promising therapeutic option for COVID-19. Almost all approved anti-SARS-CoV-2 mAb products (such as bamlanivimab plus etesevimab, casirivimab plus imdevimab, sotrovimab, and bebatelovimab) for COVID-19 clinical treatment are administered via injection.^500–503^ However, antibodies administered subcutaneously or intravenously diffuse slowly and are barely distributed in the lung where SARS-CoV-2 is concentrated. Therefore, developing inhalable antibodies, rather than increasing dosage, is a wise way to reduce costs and improve potency.^504^ Preliminary studies have demonstrated the efficacy of inhalable antibodies, including 1212C2 mAbs derived from IgM memory B cells, human single-chain variable fragment antibodies (76 clAbs), and bispecific single-domain antibodies (bn03) have been confirmed to be useful in animal models.^505–507^ However, the affinities of mAbs for specific variants and subvariants (such as Omicron and its subvariants BA, BQ, and XBB) vary dramatically, which is an obstacle to the development of mAb therapies.^191,508,509^ In contrast, mucosal secretory antibodies IgA and IgM are prone to show comprehensive affinity to variants and subvariants,^195–197^ and antibody cocktail therapies are relatively mature to enhance the curative effect of COVID-19 treatments.^510^ Therefore, the most crucial step in promoting the use of inhalable antibodies is to build a platform for inhalable antibody treatment. Maintaining the durable activity of inhalable antibodies and improving delivery systems should be considered.

To effectively promote the use of inhalable antibodies, it is essential to establish a robust platform for their development. Key considerations include maintaining the durable activity of these antibodies and optimizing delivery systems. Furthermore, it is crucial to address the potential risks associated with antibody therapies, particularly ADE. The dual nature of the Fc domain must be acknowledged; while it can enhance immunogenicity and facilitate interactions with other components of the immune system to bolster antiviral responses, it may also exacerbate infection risk through ADE. Nanobodies, which are heavy-chain-only natural antibodies that lack the Fc domain, present an alternative strategy that may circumvent the issue of ADE in therapeutic applications.^511–513^

As the COVID-19 pandemic, driven by SARS-CoV-2 variants, continues to threaten global health, ongoing monitoring and adaptation of antibody therapies are crucial. Recent analyses of conformational changes in the spike protein of various SARS-CoV-2 mutants-such as Delta, Mu, and Omicron-reveal that while the overall stability of the spike protein remains intact, local antigenic epitopes have significantly altered.^514^ The flexibility of continuous and discontinuous epitopes is vital for effective antibody recognition. The binding strengths of certain variants to hACE2 underscore the need for careful consideration in the design of next-generation vaccines and therapeutic antibodies, guiding modifications to enhance their effectiveness against emerging strains.

### Other clinical or experimental schemes

In addition to mucosal vaccines and inhalable antibodies, targeted inhibitors are also valid. An off-patent drug ursodeoxycholic acid can downregulate ACE2 by inhibiting farnesoid X receptor signaling,^515^ which prevents the entry of SARS-CoV-2. What’s more, suppressing cytokines and complement proteins is acceptable. C3, C5b, type I IFN and IL-6 inhibitors are already engaged in clinical trials.^516–519^ Although some SARS-CoV-2 inhibitors may not aim at the mucosal immune system, constructing an organoid platform for screening drugs also focuses on the relationship between the mucosal immune environment and the virus.^519,520^

Probiotic supplementation is another novel scheme to regulate microbial disorders in the gut or respiratory tract, which plays a positive role in the treatment of Parkinson’s disease,^521^ cancer,^522^ Helicobacter pylori infection,^523^ pulmonary tuberculosis,^524^ pneumonia,^525^ enteritis^526^ and Colitis.^527^ This primary care can also be employed for COVID-19 and its complications. As ingredients in nutritional supplements added to the diet, Selenium- and Zinc-Enriched *Saccharomyces cerevisiaeand* allocated 1:1 *Lactiplantibacillus plantarum* and *Pediococcus acidilactici* are related to reduced nasopharyngeal viral load and lung infiltrates;^330,528^ similarly, a Lactobacilli throat spray can also decrease viral load.^529^ Additionally, short-term dietary changes to energy-dense feast diets transiently suppress mucosal and systemic immunity by impairing CD4^+^ T-cell function.^530^

## The remodeled mucosal immune signatures of postacute sequelae of COVID-19

At least 10% of people who recover from COVID-19 will continue to experience debilitating health problems, termed long COVID-19 or postacute sequelae of COVID-19 (PASC). SARS-CoV-2 affects multiple organ systems and the sequelae of COVID-19 affect the entire body. The influence of long COVID duration can be described with respect to sex, age, and inherent disease profile.^531,532^ Long-COVID-19 duration requires more attention to reduce or avoid the long-term impact of COVID-19 and determine the relationship between COVID-19 and other diseases. Therefore, renormalizing the mucosal immunity may be a potential channel for achieving relief from long COVID.

Hundreds of biomedical findings have authenticated adverse outcomes due to the long duration of COVID-19, including pulmonary symptoms, loss of smell and taste, cerebrovascular disease, metabolic disorders, gastrointestinal disorders, reproductive dysfunction, chronic fatigue syndrome and dysautonomia which can last for years.^533–536^ Addressing PASC needs a multidisciplinary model of care,^537^ and in view of mucosal immunity, the remodeled mucosal immune signatures underlying PASC are important. Persistent virus shedding can be detected in the host even if it is not severe,^538–540^ and tissue-resident CD8+ β7Integrin + T cells and IgA redistribution in mucous membranes is one of the characteristics features of PASC, suggesting that mucosal immunity is altered because of COVID-19.^23^

Inflammation following COVID-19 triggers lasting phenotypes in immune and non-immune cells. IL-6, a cytokine significantly increasing during COVID-19, reprograms the epigenetic memory of hematopoietic stem and progenitor cells, and the progenitor cells can convey the reprogramming to their progeny innate immune cells.^541^ Excluding IL-6, IL-1β and TNF-α also elevate in PASC, these cytokines and pro-inflammatory M1 AMs retain a positive feedback loop,^542,543^ leading to chronic inflammation.^544^ In addition, adaptive immune cells also alter; CD8+ memory T cells and memory B cells converge at the infected site of the gut and lung, and their dysregulation gets involved in IBD and chronic lung impairment.^540,545^ Worse, it is found that naïve B cells and CD4 + T cells are affected by inflammation, which directly jeopardizes the neutralizing capacity of the immune system.^546,547^ To deal with catastrophic immune dysregulation, Minutolo A. et al. demonstrate that thymosin alpha 1 helps to restore the immune homeostasis of lymphocytes in PASC individuals.^548^

## Conclusion

This review underscores the intricate nature of the mucosal immune system and its significant role in responding to pathogens such as SARS-CoV-2, as well as in the development of novel therapeutic approaches. The mucosal immune system, distributed across various body surfaces, displays both commonalities and unique characteristics depending on its location. Importantly, mucosal immunity extends beyond mere protection at the body’s interfaces with the external environment, influencing broader aspects of health.

### Complexity and specificity

The mucosal immune system is characterized by its complexity, with distinct structural and functional attributes across different mucosal sites. For instance, the immune responses in the gastrointestinal tract differ markedly from those in the respiratory or urogenital tracts. Understanding these specific attributes is essential for developing targeted interventions that address the unique challenges posed by different mucosal environments. Investigating the interplay between mucosal immunity and chronic conditions, such as autoimmune disorders and gastrointestinal diseases, will be crucial in developing comprehensive approaches to disease management.

### Mucosal immune responses to pathogens

The dynamic interactions between mucosal immunity and pathogens, including SARS-CoV-2, demonstrate the adaptive nature of the immune responses. Mucosal immune signaling pathways play a pivotal role in this interaction, providing opportunities for targeted therapeutic interventions. However, the risk of adverse effects due to dysregulated immune responses necessitates a careful balance in therapeutic approaches. Insights into these pathways can guide the development of more precise and effective treatments. Future research should focus on exploring the regulatory factors that influence mucosal immune responses, such as immune cell activation, cytokine release, and signaling pathways.

### Challenges in mucosal vaccine development

One of the primary challenges in mucosal vaccine development is inducing specific immune responses while maintaining tolerance to non-pathogenic microbes. Achieving this balance requires a nuanced understanding of the immune system’s behavior in mucosal environments. Researchers must address the complexities of immune tolerance and activation to develop effective mucosal vaccines that provide robust protection without unintended side effects.

### Innovative therapies

Novel therapeutic strategies, such as nanoparticle vaccines and inhalable antibodies, have shown considerable promise in eliciting robust mucosal immune responses. These approaches offer significant potential for both preventing and treating diseases, including COVID-19. Additionally, ongoing clinical trials exploring the use of probiotics as adjunctive or personalized therapies suggest their potential role in enhancing mucosal immunity and supporting overall health.

In conclusion, the mucosal immune system represents a critical area of research with far-reaching implications for health and disease management. The ongoing investigation and development of innovative therapeutic strategies hold promise for enhancing mucosal immunity and addressing future public health challenges. Emphasizing the insights gained from mucosal immunity research will be vital for advancing our approaches to disease prevention and treatment, ultimately improving outcomes for individuals and populations.
